# Innovative inhalable dry powder: nanoparticles loaded with Crizotinib for targeted lung cancer therapy

**DOI:** 10.1186/s12885-025-15015-w

**Published:** 2025-10-07

**Authors:** Faiza Naureen, Yasar Shah, Maqsood Ur Rehman, Fazli Nasir Fazli Nasir, Abdul Saboor Pirzada, Jamelah Saleh Al-Otaibi, Maria Daglia, Haroon Khan

**Affiliations:** 1 Department of Pharmacy, Abdul Wali Khan University Mardan, 23200 Mardan, Pakistan; 2 Department of Pharmacy, University of Malakand, Lower Dir, Chakdara, Pakistan; 3 Department of Pharmacy,, University of Peshawar, Peshawar, Pakistan; 4 Department of Chemistry, College of Science, Princess Nourah Bint Abdulrahman University, P.O. Box 84428, Riyadh, 11671 Kingdom of Saudi Arabia; 5 Department of Pharmacy, University of Napoli Federico II, Via D. Montesano 49, NA, 80131 Naples Italy; 6 International Research Center for Food Nutrition and Safety, Jiangsu University, Zhenjiang, 212013 China; 7 Department of Pharmacy, Korea University, Sejong, 20019 South Korea

**Keywords:** Lung cancer, Crizotinib, Pulmonary delivery, Optimization, Polymeric nanoparticles, Inhalable dry powder

## Abstract

Crizotinib is a targeted therapy for metastatic non-small cell lung cancer (NSCLC) that is ALK- or ROS1-positive, as well as for conditions such as ALK-positive anaplastic large cell lymphoma and inflammatory myofibroblastic tumor. However, the associated toxicity poses a significant challenge to its use. To mitigate this issue, a novel dry powder inhalation formulation was developed using Crizotinib-loaded polyethylene glycol-based polymeric nanoparticles (NPs). These nanoparticles were created through a nanoprecipitation approach and improved employing central composite design. The capabilities of the formulation were assessed with Anderson Cascade Impactor, revealing a fine particle fraction of 56.2% and a mass median aerodynamic diameter of around 1.5 μm, indicating appropriate aerodynamic characteristics for inhalation. Key properties of the optimized nanoparticles included Encapsulation efficiency (82.3%, Zeta potential (-31.9 mV), Particle size (167 nm), Polydispersity index (0.462) and Release efficiency (60.6%) In vitro studies indicated that the polymeric nanoparticles exhibited greater anticancer activity compared to free Crizotinib. Additional characterization using techniques like XRD, DSC, FTIR, and SEM confirmed that the polymeric nanoparticle formulation has promising physicochemical properties, suggesting it could enhance local drug delivery and efficacy in lung cancer treatment while potentially reducing systemic toxicity.

## Introduction

Approximately 20% of all cancer deaths (1.6 million) are attributed to lung cancer (LC), making it one of the lethal cancers for both men and women [1]. Non-small cell lung cancer (NSCLC) and small cell lung cancer (SCLC) account for 85% and 15% of all cases of lung cancer, respectively. The low 5-year survival rate of 15% for NSCLC its often-delayed detection is largely due to ineffective therapy and late-stage diagnosis, emphasizing the urgent need for improved treatment strategies.

Cancer treatment for lung cancer primarily include chemotherapy, radiation therapy, and surgical resection [2]. Among these, chemotherapy is the most widely used approach; however, its effectiveness is limited by several challenges. These include inadequate selectivity, which can lead to damage to healthy tissues, significant side effects such as nausea, fatigue, and immunosuppression, and the emergence of drug resistance over time. These issues significantly hinders the overall success of lung cancer treatment [3].

Molecular targeted therapies represent a promising advancement in addressing the lack of specificity in conventional chemotherapy. One of these targets that is a receptor tyrosine kinase that is inhibited by crizotinib. Crizotinib, a receptor tyrosine kinase inhibitor, specifically targets tumors that are anaplastic lymphoma kinase (ALK) or ROS1-positive. This drug is also utilized for treating ALK-positive anaplastic large cell lymphoma (ALCL) and inflammatory myofibroblastic tumor (IMT) [4]. By inhibiting ALK signaling pathways, crizotinib promotes apoptosis in cancer cells and inhibits tumor proliferation [5]. However, its clinical application faces several obstacles, including low water solubility, poor bioavailability (less than 44%), and significant toxicities such as diarrhea, nausea, vomiting, stomatitis, anorexia, skin rash, and interstitial lung disease [6]. Additionally, achieving adequate drug concentrations at tumor sites poses a considerable challenge, underscoring the need for effective drug delivery systems [7, 8].

Inhalable therapies offer several advantages over systemic administration, including noninvasiveness, improved patient compliance, and avoidance of first-pass metabolism, which can degrade the drug before it reaches the systemic circulation. By delivering high concentrations of anticancer drugs directly to lung tumors, aerosolized chemotherapy may reduce the overall dosage required and associated systemic side effects. Furthermore, inhalation can minimize treatment interruptions that may lead to tumor cell repopulation, thereby enhancing treatment efficacy [9, 10]. Despite the potential benefits of inhalable cancer treatments, no inhalation chemotherapy has yet been commercially available. This gap is partly due to the rapid absorption of administered drugs and the quick clearance of unbound drugs through phagocytosis in the alveolar region, which limits their therapeutic effectiveness [11].

Nanomaterials have shown significant promise in the development of effective drug delivery systems for respiratory diseases. Their ability to prolong drug release, reduce dosing frequency, and enhance the retention time of therapeutic agents in the lungs makes them particularly attractive for inhalable [12]. Various nanocarriers, including liposomes, polymeric micelles, macromolecular conjugates, and nanoparticles (NPs), have been investigated for targeted delivery of chemotherapy drugs to tumor sites.

Polymeric nanoparticles are favored for their low toxicity, biodegradability, and intrinsic biocompatibility. They can achieve high drug payloads, encapsulate both hydrophilic and lipophilic drugs, protect sensitive medications from degradation, and are relatively easy to prepare and manufacture on a large scale [13]. Their ability to provide controlled and sustained release of drugs is particularly advantageous in cancer treatment. Surface modification of nanoparticles with hydrophilic polymers like polyethylene glycol (PEG) can help evade the host’s immune system, reducing recognition and uptake by immune cells [14]. This modification enhances the circulation time of nanoparticles in the bloodstream, allowing for greater accumulation at tumor sites [15]. PEG-based polymeric nanoparticles were selected for this study owing to their distinct physicochemical and biological advantages over other nanocarriers employed for pulmonary delivery. PEGylation imparts a hydrophilic surface coating that reduces opsonization by alveolar macrophages and thereby prolongs pulmonary residence time. Compared with liposomes and solid lipid nanoparticles, which often suffer from stability issues such as lipid oxidation or fusion during storage, PEG-based polymeric systems exhibit superior physical stability. Furthermore, PEG polymers can be tailored to achieve controlled drug release and improved mucus penetration in the respiratory tract, which are critical for effective local drug delivery. In contrast to cationic carriers such as chitosan nanoparticles that may induce local irritation or cytotoxicity, PEG-based systems are biocompatible and less likely to trigger inflammatory responses. These characteristics make PEG-based polymeric nanoparticles an attractive platform for developing inhalable formulations of anticancer drugs like crizotinib.

Methods for pulmonary drug delivery include nebulizers, soft-mist inhalers, dry powder inhalers (DPIs), and pressurized metered-dose inhalers (pMDIs). Among these, DPIs are becoming increasingly popular due to their stability for long-term storage, suitability for drugs with low water solubility, and their portability and ease of use. DPIs do not require a propellant, making them environmentally friendly and cost-effective [16, 17].

Crizotinib-loaded targeted polymeric nanoparticles were developed through an optimized process, and their physicochemical characteristics and release patterns were rigorously evaluated. Following optimization, the selected formulation was used to create the DPI dosage form, with performance evaluated using the Anderson Cascade Impactor [18, 19]. This study aims to establish a viable inhalable delivery system for Crizotinib, potentially improving therapeutic outcomes for lung cancer patients while reducing its toxicity.

## Materials and methods

### Materials

Crizotinib (purity 99.9%), purchased from Shaanxi Dideu Medichem Co., Ltd., China, PEG (MW 6000), Sigma Aldrich; Stearic acid, Sigma Aldrich; Methanol (purity > 99.9% sigma), Ethanol (Scharlau, reagent grade 99.8%), potassium dihydrogen phosphate were purchased from Sigma Aldrich (St. Louis, MO, USA), Ortho-phosphoric acid (ScharlauChemie, Barcelona, Spain), Sodium Bicarbonate (NaHCO3), 99.95% purity (Fluka), Potassium Chloride, Sodium Chloride Di-sodium Hydrogen Phosphate, Di-potassium hydrogen phosphate, (Scharlauchemie, Spain), Tween 80 (Sigma), Mannitol was purchased from Merck Chemical Company, Dialysis membrane, (MWCO; 12-14 kDa); (Sigma-Aldrich), distilled water [Millipore ultrapure water sys. (Milford, USA)].

### Method

#### Computational analysis for compatibility study of drug with excipients

PubChem was used to retrieve the chemical structure Crizotinib, Oleic Acid, PVP, Palmitic acid, PEG and Stearic Acid. The YASARA-Structure programme was utilised to do energy minimization and optimization of all retrieved structures (20). This Programme gives better structure interaction identification between drug and excipient [20, 21].

Although Crizotinib was only used as a ligand (guest) structure in the molecular docking simulations, the structures of all other components Oleic Acid, PVP, Palmitic acid, PEG, Stearic Acid polymers were considered as probable receptors (host) and ligands (guest) [22, 23]. To ensure that all potential drug interactions were examined, the grid box was adjusted molecular docking simulations by utilizing AutoDock Vina in PyRx for. All co-polymer and drug complexes were visualized utilizing the Discovery Studio Visualizer [24–26].

#### Preparation of nanoformulation

**Fig. 1:**
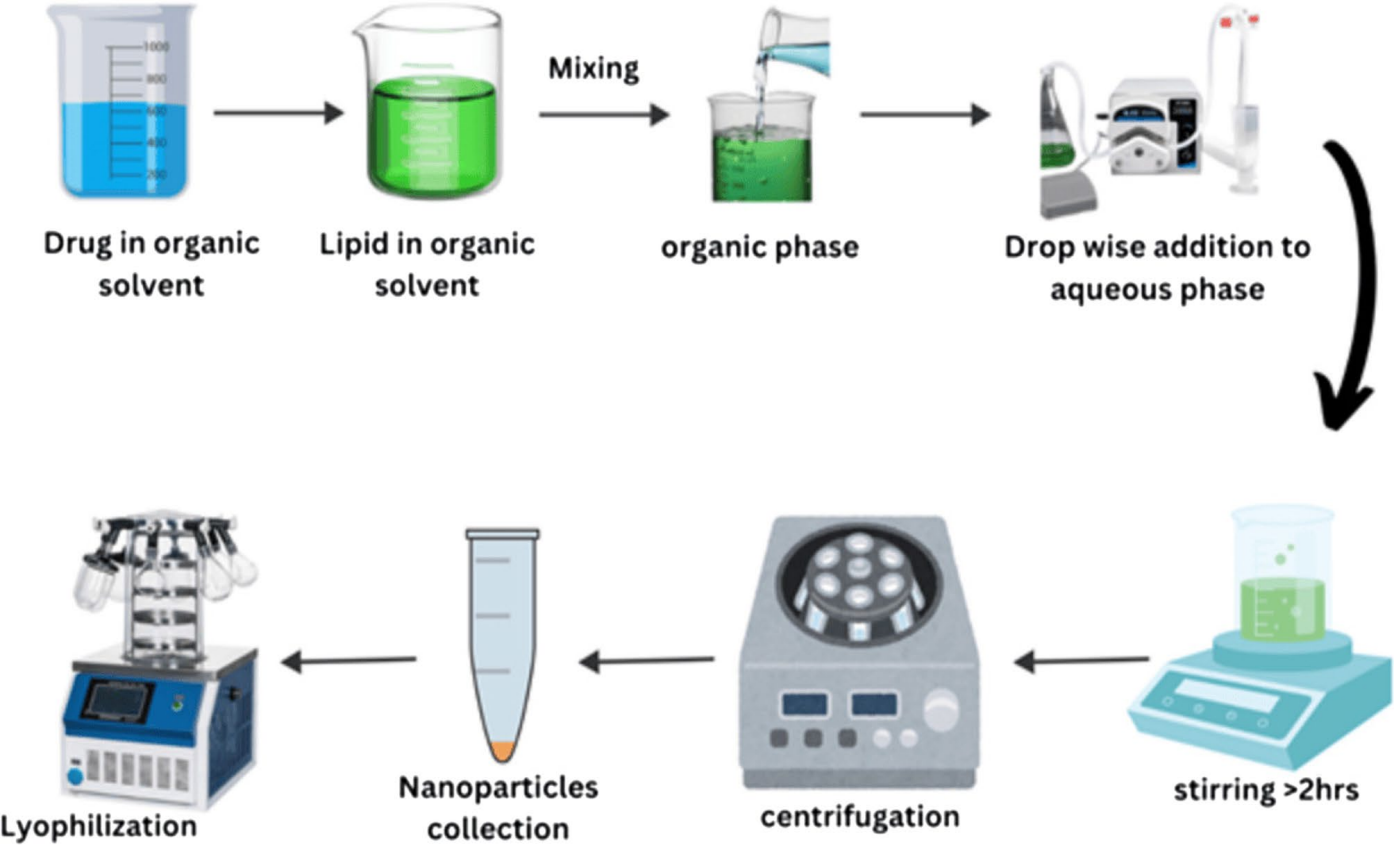
Crizotinib Nanoparticles preparation

The process of nano-precipitation was employed to create polymeric nanoparticles loaded with crizotinib. Process is described with help of Fig. 1. Lipid was melted above its melting point and was dissolved in organic solvent ethanol. Lipids were first melted and dissolved in an organic solvent to achieve molecular dispersion, which facilitated homogeneous mixing with the polymer and drug, ensured efficient encapsulation of the hydrophobic drug, and prevented crystalline aggregation during nanoparticle formation. Drug was then dissolved in ethanol and was added to lipid solution to form organic phase. Drug with different percent ratio (0.2, 0.5,1 and 2%) was tried to get perfect formulation. Polymer was dissolved in deionized water to form aqueous phase with constant stirring above 10,000 rpm. Temperature of the solution was maintained at 75 °C. Surfactant was added to aqueous phase with constant stirring of the solution [27]. Afterward, the organic phase was added drop wise to aqueous phase with rate of 1 ml/min using a peristaltic pump (Model NE-9000B) while keeping aqueous phase continuously stirred. Stirring was done for 2 h to evaporate organic phase. Subsequently, the prepared nanoparticles in nano-emulsion were collected by centrifugation for 30 min at 8,944 g rcf with temperature 5 °C. All nanoformulation were prepared in triplicate. The nanoparticles were triple-washed with distilled water [28] during centrifugation to remove excess surfactant and unencapsulated drug followed by gentle resuspension of pellets in fresh medium to minimize loss. Afterward nanoparticles were freeze-dried [29].

#### Experimental design and analysis

Response surface model (RSM)/Central composite design (circumscribed) was used to study the influence of each of the varied factors. The effects caused by independent factors on the dependent factors was studied by optimizing method used in RSM [29, 30]. In the current study, the four selected independent factors i.e. the amount of polymer, the amount of lipid, percentage of surfactant and stirring time were selected. It is given in Table 1. The two factorial levels along with three center points were coded as −1, 0, and + 1 for low, center, and high levels were used in the study.

**Table 1:** Factor levels in central composite design

Independent Factors	Levels		
	−1	0	1
Amount of polymer mg/ml	5	7.5	10
Amount of lipid mg/ml	10	15	20
Percentage of surfactant %	1	2	3
Stirring time (min)	30	75	120

The Design Expert v. 13.0 program (StatEase, Minneapolis, MN, USA) recommended a total of 17 runs, and the responses were determined by Particle size (R1), zeta potential (R2), and entrapment efficiency (R3) and in vitro drug release (in 12 h) (R4). Table 2 had shown different factors levels. In order to produce a polynomial equation that shows estimations of coefficients for statistically significant individual factors (β#) and significant two-factor interactions (β##), ANOVA was used to assess the importance and effect of independent factors and their two-factor relations on each of the responses [31]. Following the completion of the trials listed in Table 2, these coefficients are based on the factor levels and the reaction to each set of.

factor levels. Here is the polynomial equation:

$$\mathrm Y={\mathrm\beta}_{0\;}+\sum_{\mathrm i=0}^{\mathrm n}\;{\mathrm\beta}_{\mathrm i}\;{\mathrm x}_{\mathrm i}+\sum_{\mathrm i=0}^{\mathrm n}\;{\mathrm\beta}_{\mathrm{ii}}\;\mathrm x_{\mathrm i}^2+\sum_{\mathrm i\neq\mathrm i=1}^{\mathrm n}\;{\mathrm\beta}_{\mathrm{ij}}\;{\mathrm x}_{\mathrm i}\;{\mathrm x}_{\mathrm j}$$

Y stands for the suggested answer, β0 for the intercept, n for the number of components examined, and βi, βii, and βij for the linear (main effect), quadratic, and interaction model coefficients, respectively. The levels of the independent variables are similarly described by Xi and Xj [32].

**Table 2:** Real factor levels for three center points in a central composite design

		Independent variables			
Regular Run Order	Irregular Run Order	Amount of polymer (mg/ml)	Amount of lipid (mg/ml)	Percentage of surfactant (%)	Stirring time (min)
1	10	11.7	15	2	75
2	8	10	20	3	120
3	12	7.5	23.4	2	30
4	14	7.5	15	3.68	75
5	7	5	20	3	120
6	2	10	10	1	120
7	5	5	10	3	30
8	9	3.29	15	2	75
9	13	7.5	15	0.31	75
10	4	10	20	1	75
11	1	5	10	1	30
12	17	7.5	15	2	15
13	15	7.5	15	2	75
14	11	7.5	6.59	2	120
15	6	10	10	3	120
16	3	5	20	1	30
17	16	7.5	15	2	165

### Assessment of polymeric nanoparticles loaded with Crizotinib

To redispersed the particles by making stock solution of 2 mg/ml in distilled water and then further diluted with distilled water at 0.2 mg/ml concentration for PDI, Particle size and 0.1 mg/ml for Zeta potential assessment. For in vitro release 2 mg/ml stock was prepared. All physicochemical assessments experiments were performed in triplicate and *±* SD are given in Table 4.

#### Size of particles and Poly-Dispersibility index (PDI)

Zeta-Sizer (Nano ZS-90, Malvern Instrument, and Malvern, UK) evaluated the PS and PDI of nano-formulations. Every measurement was made in triplicate, and each sample’s mean ± SD was computed [33].

#### Zeta potential

Using Zeta-Sizer (Nano ZS-90, Malvern Instruments, Malvern, UK), the ZP of the nano formulations. All measurements was done in triplicate, the mean ± standard deviation (SD) was determined [34].

#### Encapsulation efficiency (EE) and drug loading (DL)

The drug-loaded nanoparticles were separated from the aqueous medium by centrifuging the nanoparticles for 30 min at 2,236 g rcf and 5 °C (Centurion^®^ Scientific, UK). Using a UV spectrophotometer (Shimadzu^®^, Japan), the quantity of free medication in the supernatant was measured. The % EE and DL% were then computed using the following formula given in equations.

$$\text{EE} \%=\frac{{\text{Total amount of drug added}}-\text{Unloaded Drug}}{\text{Total amount of drug }}\times 100$$

$$\text{DLC}\%=\frac{\text{Total quantity of drug in Nanoparticles}}{\text{Quantity of Drug}+\text{Quantity of Excipients}}{\times 100}$$

#### In-vitro drug release

In accordance with ICH recommendations, the release profile of the drug from the improved nano-formulation was evaluated. The nano-formulation were examined in triple times. The *in -vitro* drug release pattern of crizotinib from polymeric nanoparticles was ascertained by means of a dialysis membrane (Dialysis Tubing-Visking MWCO: 12-14 kDa). In order to create sink conditions, about 1 milliliter of each formulation was placed in a dialysis bag and submerged in phosphate buffer solution (PBS, 500 ml pH = 7.4) that included 2% Tween 80. The temperature was maintained at 37 ± 0.2 °C while the release medium was constantly agitated at 100 rpm. One milliliter of the sample was taken out at a predetermined interval and replaced with an equivalent volume of new medium. The drug’s concentration was then measured using spectrophotometry at 270 nm [35].

### Characterization of lipid polymer hybrid Nano-particles loaded with Crizotinib

#### Scanning electron microscope (SEM)

Drug-loaded nanoparticles were characterized by form and surface morphology using SEM (JSM-5910, Jeol, Japan) operated at an accelerating voltage of 15 kV for analysis [36]. The lyophilized nanoparticles were dispersed over the sticky carbon tape that was fastened to the stub in preparation for the SEM examination. Gold (Au) was applied to the surface of the nanoparticles for approximately ninety seconds under vacuum using a coater (Argon Sputtering, SPI Module Control). The prepared sample was then examined using a scanning electron microscope (SEM) at various magnifications, and pictures were captured [37].

#### Power X-RAY diffractometry (XRD)

The XRD pattern of the pure drug, and optimized formulation with 1:1 physical mixing of drug with polymer, surfactant and stearic acid formulation was analyzed in an angle range (2θ) of 10°–40 using an X-ray diffractometer (JDX-3532, JEOL, Japan) [38].

#### Differential scanning calorimetry (DSC)

Differential scanning calorimetry (DSC) was used to evaluate the drug’s thermal characteristics throughout the conversion to an optimized nanoparticles formulation utilizing a universal V2.4 F TA Instrument USA. Within the metal pans, at least 5 milligrams of the sample were sealed. Ten Co/min was maintained as the heat transmission rate. The temperature range in the DSC was 80 °C to 330 °C, and a nitrogen environment was also preserved [39].

#### Fourier transform infrared spectroscopy (FTIR)

One of the optimized nano-particles formulation was used for analysis in FTIR. The Shimadzu FTIR spectrophotometer (Shimadzu Corporation, Kyoto, Japan) was used for the investigation at scanning range 4000–400 cm⁻¹, Resolution: 4 cm⁻¹ average 32 scan.

FTIR spectra for pure drug and optimized formulation was compared after analysis by FTIR [40, 41].

### Stability study

The optimized LPHNPs formulation was split into two portions and kept for four weeks at room temperature (25^0^ ± 2 ~C) and chilled (4^0^ ± 3 ~C). For both temperatures, average particle size, PDI and encapsulation effectiveness were determined on various time gap [42, 43].

### Preparation of inhalable dry powder (DPI) containing Crizotinib based polymeric nano-particles

Making a DPI that includes polymeric nano-particles (NPs) were created by freeze-drying process. Optimized NPs (equal to 50 mg crizotinib) was then physically admixture with 0.5% mannitol and finished product was formed. Then that finished product was kept at room temperature in a desiccator until analysis [43, 44].

#### In vitro powder deposition study

Lung deposition of dry particles was assessed using the Anderson Cascade Impactor (ACI, Copley Scientific, UK). Tween 80 (1% w/v in ethanol) was used to wet the collection plates in order to stop particle bounce and re-entrainment [45]. A mouthpiece adaptor was used to connect the induction port to the Handihaler device (Boehringer Ingelheim, Germany) in order to produce an airtight seal. Low Capacity Pumps Models LCP5, Copley Scientific Ltd., UK, connected ACI to a vacuum source via a critical flow controller (TPK 2000) [35, 46]. 

A firm gelatin capsule containing 25 mg of each powdered nano-composite was placed inside the Aerolizer inhaler. The dry powder was aerosolized into ACI for 4 s at a flow rate of 60 L/min after the capsule burst. In the end, the mouthpiece adapter, throat, and eight plates were cleaned with one milliliter of water.

The drug was extracted using dichloromethane 2 ml and measured spectrophotometrically at 270 nm after the mouthpiece adapter, throat, and eight plates were cleaned with 1 mL of water.

To determine the emitted dose (ED), the entire quantity of crizotinib extracted from the mouthpiece, induction port, pre-separator, and all phases was utilized. The deposited dosage (DD) is the total mass of crizotinib deposited on stages 0 through F. Equation 4 was used to express the percentage dispersed (PD) of the ejected dose (ED) based on the total dosage (TD). The percentage of DD depending on the TD was represented as the percent inhaled (PI) using Eq. 5. For stages 2 through F, stages 3 through F, and stages 3–7, the fine particle doses (FPD) were calculated at two distinct levels: FPD < 3 μm and FPD < 5 μm [47]. The first level showed particles that were usually deposited on the deep lung. The second level represented particles deposited on the peripheral lung. Level three was that level, which excluded particles that were likely to be inhaled (less than 500 nm). Equation was utilized to represent the fine particle fraction (FPF) as a proportion of FPD to ED. Equation 7 was utilized to represent the respirable fraction (RF) as the percentage of FPD to the DD.

$$\text{Percent dispersed (PD)}\%=\text{ED/TD}\times100$$

$$\text{Percent inhaled (PI)}\%=\text{DD/TD}\times100$$

$$\text{Fine Particle Fraction (FPF)}\%=\text{FPD/ED}\times100$$

$$\text{Respirable Fraction (RF)}=\text{FPD/DD}\times100$$

In vitro deposition graph was used to display the cumulative particle distribution functions that were derived from the ACI. Aerosolization performance testing using the Andersen Cascade Impactor was conducted in three independent replicates for each formulation, and the average values were used for data analysis. The averages and standard deviations of the aerosolization and inhalation parameters were derived from triplicate experimental runs [48].

## Results and Discussion

### Analysis by computation

Docking of molecules is an indispensable modelling tool that reveals the interactions between the receptor (host) and ligand (guest). This method permits the visualization of binding regions for donor and changes inside a host. Most of the time, the simulation of molecular docking offers information regarding the way of the drug orientation within a binding site (known as the “pose”) and an evaluation of the detected pose’s binding affinity as a score value. To ascertain the binding energy of a particular ligand orientation, the AutoDock-VINA technique employs a machine-learning approach that combines the benefits of knowledge-based potentials with empirical scoring functions. As indicated in Table 3 AutoDock Vina was utilised to compute the comparative binding free energies between Crizotinib and a variety molecule of polymers and co-polymeric Oleic Acid, PVP, Palmitic acid, PEG, Stearic acid component.

**Table 3:** Calculation of binding energies for a variety of Co-polymeric systems, including oleic acid, PVP, palmitic acid, PEG, stearic acid and Crizotinib

Host	Ligand	Binding Energy	Binding Interaction
Crizotinib	Oleic Acid	−2.6	
Crizotinib	PVP	−2.2	
Crizotinib	Palmitic acid	−2.1	
Crizotinib	PEG	−2.3	
Crizotinib	Stearic Acid	−3.7	
Oleic Acid	Crizotinib	−2.7	
PVP	Crizotinib	−2.1	
Palmitic acid	Crizotinib	−3.5	
PEG	Crizotinib	−3.7	
Stearic Acid	Crizotinib	−3.3	

The binding free energies between a host molecule (Oleic Acid, PVP, Palmitic acid, PEG, Stearic acid) and a guest molecule (Crizotinib) can be used to determine the strength of their interactions. Compared to looser contacts/binding, tighter connections between crizotinib and polymer may result in strong drug–polymer network and a high extended release of drug profile. The tying free energy table demonstrates that the single polymeric form has reduce tendency for binding than the double polymer molecules. Table 3 shows the binding energies between Crizotinib and several co-polymer complexes. Binding energy indicates the strength of the interaction between Crizotinib and the co-polymer, with lower values signifying weaker binding. When comparing the Binding Affinity with Crizotinib as host, all ligands except PVP exhibit similar binding energies with Crizotinib (around − 2.1 to −3.7 kcal/mol). This suggests that these ligands have comparable affinity for Crizotinib. PVP has the weakest binding affinity with Crizotinib (−2.2 kcal/mol). This indicates that PVP interacts less favorably with Crizotinib compared to other ligands. When Comparing the Binding Affinity with Crizotinib as Ligands: Crizotinib shows a stronger binding affinity with stearic acid (−3.7 kcal/mol) compared to other ligands when it acts as a ligand itself. This suggests that the interaction between stearic acid and Crizotinib is energetically more favorable when Crizotinib acts as the ligand. Moreover, binding energy analysis revealed favorable interactions between crizotinib and the lipid–polymer matrix, suggesting a stable encapsulation environment. Strong drug–excipient interactions contribute to improved nanoparticle stability by minimizing premature drug leakage, while balanced binding energy facilitates controlled and sustained release under physiological conditions. These results, in agreement with the vacuolization study, indicate that the hybrid structure effectively maintains particle integrity, thereby supporting the potential of the formulation for pulmonary delivery. This study offers a preliminary insight into the binding interactions between Crizotinib and various polymeric component.

### Nanoparticles optimization on basis of components and levels included in DOE

#### Design of experiment

An experimental design was established to carry out the investigation based on preliminary investigations. Crizotinib-loaded lipid polymer hybrid nanoparticles were optimized by the nano-precipitation process using central composite design. Particle size, Particle potential and entrapment efficiency and in vitro drug release were the four variables on which the influence of four independent factors—the amount of polymer, the amount of lipid, and the percentage of surfactant used, stirring time—was examined. Drug percent ratio (0.5%) was selected for best results. Seventeen trials in all were conducted as recommended by the Design Expert^®^ program [49]. The steps were carried out three times, and the results are displayed in Table 4.

**Table 4:** Run parameters and responses of central composite design

Std	Run	Factor A Polymer conc. (mg/ml)	Factor B Lipid conc. (mg/ml)	Factor C Surfactant percentage (%)	Stirring Time (min)	Response 1 Particle size (nm)	Response 2 Zeta potential (nm)	Response 3 Entrapment efficiency (%)	Response 4 In vitro drug release in 12 h. (%)
1	10	11.7	15	2	75	129 ± 16.0	−18.5 ± 0.6	63.3 ± 0.04	51.8±
2	8	10	20	3	120	167 ± 17.7	−31.9 ± 0.7	82.3 ± 0.05	60.6 ± 0.6
3	12	7.5	23.4	2	30	212 ± 22.9	−32.2 ± 0.5	80.0 ± 0.02	56.0±
4	14	7.5	15	3.68	75	168 ± 15.5	−32.7 ± 1.0	68.4 ± 0.06	47.7 ± 0.6
5	7	5	20	3	120	232 ± 20.0	−29.3 ± 0.8	61.7 ± 0.08	42.7 ± 0.7
6	2	10	10	1	120	179 ± 6.5	−20.8 ± 0.6	59.7 ± 0.02	51.1±
7	5	5	10	3	30	225 ± 14.3	−26.5 ± 0.6	68.5 ± 0.10	54.3 ± 0.7
8	9	3.29	15	2	75	282 ± 24.7	−18.3 ± 0.9	49.9 ± 0.04	45.2 ± 0.6
9	13	7.5	15	0.31	75	244 ± 10.9	−25.5 ± 0.6	59.8 ± 0.06	47.2 ± 1.1
10	4	10	20	1	75	187 ± 12.7	−26.8 ± 0.7	76.2 ± 0.08	52.8 ± 0.9
11	1	5	10	1	30	270 ± 25.0	−23.1 ± 0.9	62.3 ± 0.05	51.4 ± 1.3
12	17	7.5	15	2	15	206 ± 18.4	−19.6 ± 1.1	68.6 ± 0.02	54.4 ± 1.3
13	15	7.5	15	2	75	206 ± 18.4	−19.6 ± 1.1	68.8 ± 0.02	56.2 ± 0.6
14	11	7.5	6.59	2	120	199 ± 17.4	−24.8 ± 0.6	72.6 ± 0.09	50.5 ± 0.8
15	6	10	10	3	120	134 ± 9.82	−23.4 ± 0.8	62.4 ± 0.10	50.5 ± 0.8
16	3	5	20	1	30	277 ± 7.48	−24.1 ± 0.7	54.1 ± 0.13	49.4 ± 0.5
17	16	7.5	15	2	165	206 ± 18.4	−19.6 ± 1.1	68.6 ± 0.02	55.6 ± 0.6

### Analysis of findings

Four responses i.e. particle size, zeta potential, entrapment efficiency and in vitro percent drug release were estimated by Response surface model. For this plan of work the central composite design (CCD) was used for designing seventeen formulations. Four variables are given in the Table 4 with different approaches. ANOVA, regression coefficient, R2 value, and lack of fit are also included in Table 5 for statistical analysis.

**Table 5:** Findings/Results from analysis of variance (ANOVA) and regression coefficient

Source	Particle size (nm)	Zeta potential (mv)	%Entrapment Efficiency	In vitro % drug release in 12 h
β^o^	205.9	−19.70	68.64	56.23
A	−1.61	−0.0173	0.2658	0.1356
B	0.1375	−2.25	0.1485	0.1357
C	−0.7885	−2.08	0.1596	0.0070
D	0.0038	−2.06	0.0059	0.0386
AB	0.0511	−1.34	0.3637	0.0485
AC	−0.1306	0.1125	−0.0265	0.2318
AD	0.0340	0.0340	0.0012	0.2154
BC	0.0216	−0.5375	0.0221	−0.0364
BD	−0.0405	−0.0405	0.0432	0.1926
CD	0.0380	−0.0380	−0.0214	−0.2911
A^2^	−0.0992	0.4458	−0.2749	−0.1888
B^2^	−0.0313	−3.13	0.1919	0.0557
C^2^	−0.0229	−3.34	−0.1011	−0.2133
D^2^	−0.0021	−3.42	−0.0032	−0.0293
p-value	<0.0001	<0.0001	0.0012	0.0029
F-value	722.40	577	823	345
R^2^	1.0000	0.9994	0.9989	0.9996
Adjusted R^2^	1.0000	0.9987	0.9975	0.9967
Predicted R^2^	0.9999	0.9963	0.9909	0.9952
Lack of Fit	0.9988	0.7085	0.8651	0.5044

Lack of fit indicates the model’s fitness (*p* > 0.1). The model’s suitability for accurately predicting the impacts of the four variables was demonstrated by that. The impacts of variables on particle size, zeta potential, entrapment efficiency, and in vitro % drug release are fully explained by providing surface reaction graphs of all responses.

#### Analysis of Drug-loaded lipid polymer hybrid nanoparticle particle size

The physico-chemical characteristics of lipid polymer nanoparticles for pharmacy practice are significantly influenced by their particle size and particle dispersion index (PDI). Particle size is the most crucial aspect in the absorption of nanoparticles and their intracellular mobility. Particle size (< 200) with PDI (0.1 to 0.4) indicates significant extended permeability and retention. This effect-induced tumor tissue penetration. The results of the trials showed that the amount of surfactant (tween 80) has an indispensable effect on the particle size both alone (p-value of < 0.0001) and in combination with the polymer (*p*-value of < 0.0001), lipid (*p*-value 0.0013) stirring time (*p*-value 0.001). An increase in surfactant concentration resulted in a marked reduction in particle size and polydispersity index (PDI). This effect can be attributed to the ability of surfactant molecules to lower interfacial tension between the aqueous and organic phases during emulsification. Adequate surfactant coverage stabilizes the interface, prevents coalescence of droplets, and produces smaller, more homogeneous nanoparticles.

#### (a) graphical analysis

**Fig. 2:**
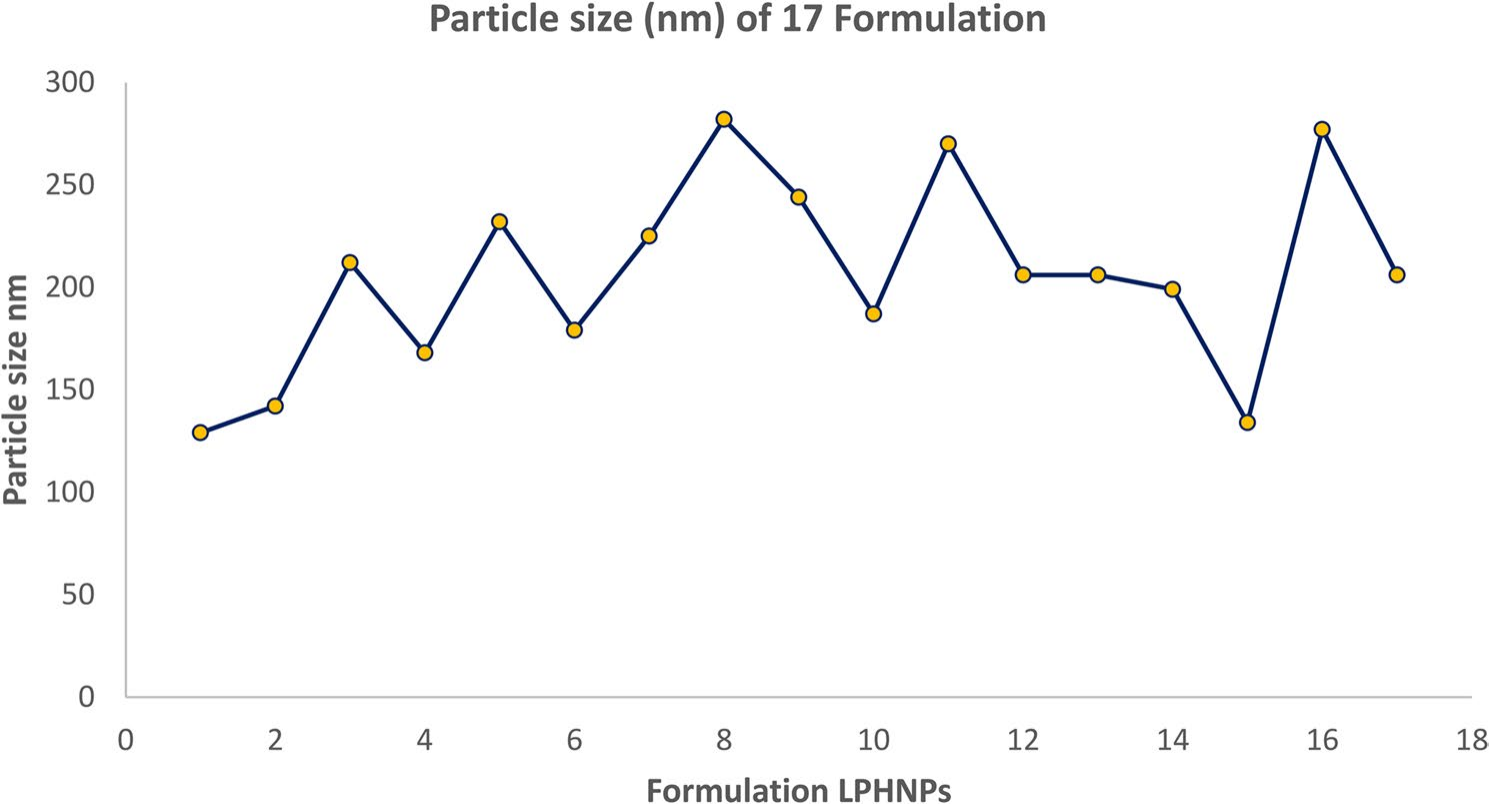
Graphical representation of different Particle size of 17 Nanoformulation

The elements/variables that have a significant impact on the size of particles were identified by analyzing the data from the trials using graphical analysis. The result of different amount of polymer, lipid and surfactant and stirring time showed high effects on size of different nanoparticles-based formulation. The different size ranges of formulation are determined in Fig. 2.

Particle size of all formulation is determined by CCD. Different particle size of different formulation showed that different concentration of polymer, lipid, surfactant and stirring time had positive impact over it. The ratio of polymer and lipid 1:2 with 3% surfactant along with 2 h stirring showed acceptable particle size.

Four independent factors’ significance levels were determined using a central composite design. Multiple regression analysis and the model’s coefficient were used to assess the significance of the four factors’ levels. Four independent variables showed significant linear, interaction, and quadratic effects (*p* < 0.05).

Particle size independent variables exhibited an excellent regression coefficient (R2) of 0.999 and were quadratic. Design Expert software version.13, which is provided below, was used to produce the final particle size prediction equation;

$$\begin{aligned}\bf\text{Y}1=&\bf+205.6-1.61\text{A}+0.1375\text{B}-0.7885\text{C}\\&\bf+0.0038\text{D}+0.0511\text{AB}-0.1306\text{AC}\\&\bf+0.0340\text{AD}+0.0216\text{BC}-0.0405\text{BD}\\&\bf+0.0380\text{CD}-0.0992\text{A}^2-0.0313\text{B}^2\\&\bf-0.0229\text{C}^2-0.0021\text{D}^2\end{aligned}$$

**Fig. 3:**
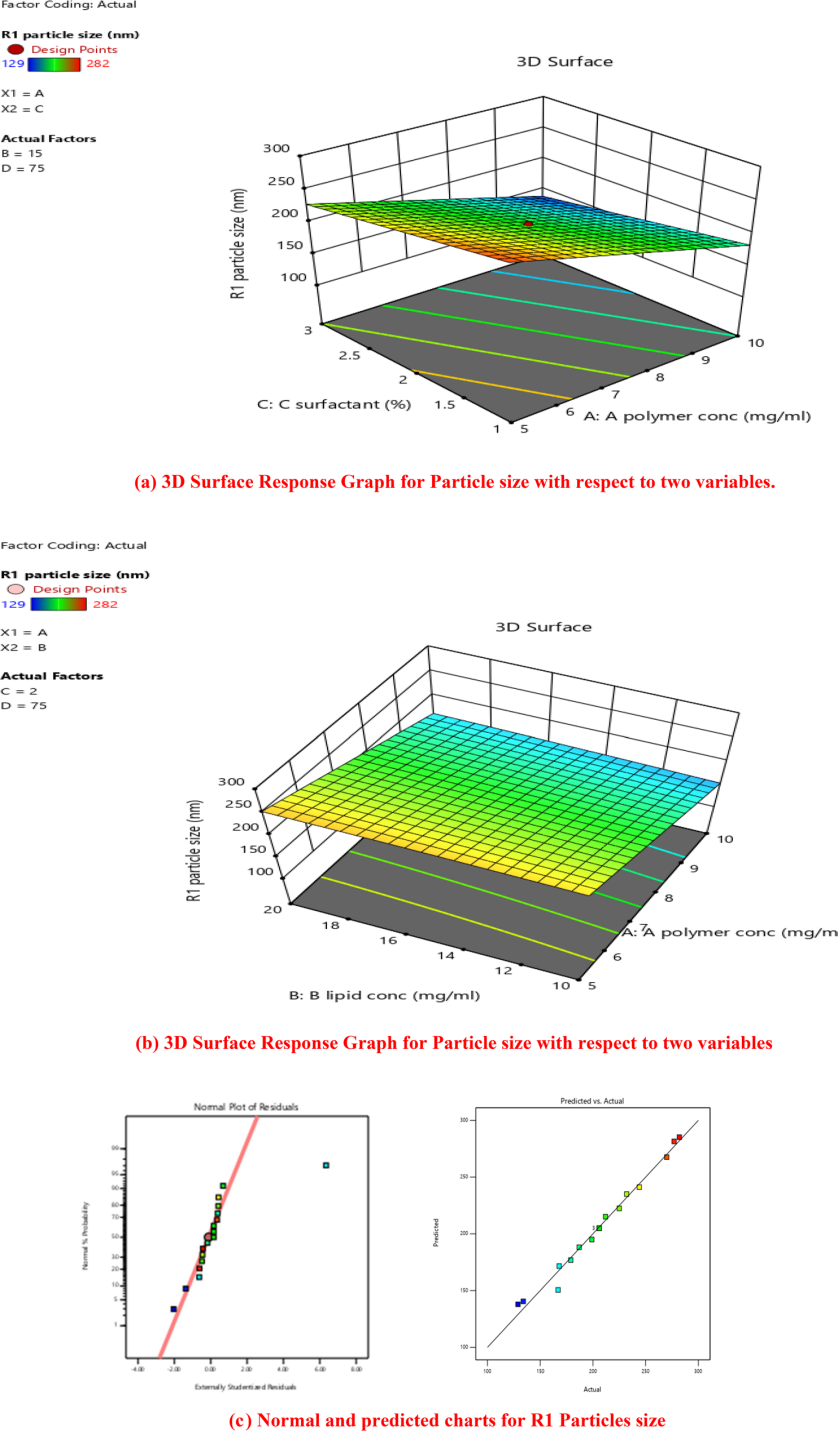
(**a**) 3D Surface Response Graph for Particle size with respect to two variables. (**b**) 3D Surface Response Graph for Particle size with respect to two variables. (**c**) Normal and predicted charts for R1 Particles size

Three-dimensional response surface contour plots (surface response graphs) were used to depict the optimal level impacts of four independent factors on particle size. Four independent factors were shown to have a substantial impact on particle size (Fig. 3 (a) and (b)).

For certain amounts of each element, predictions regarding the reaction may be made using the equation expressed in terms of coded factors. By default, the components’ high and low values are denoted by the numbers + 1 and − 1, respectively. By comparing the factor coefficients, the coded equation may be used to determine the relative importance of each component [49].

The interaction between Factor A (polymer) and Factor B (lipid) was given in 3D response surface plot. The plot clearly showed that, while operating at low polymer, there was a significant variance in the size of particle when going from low polymer (5 mg/ml) to high polymer (10 mg/ml). When operating at a high polymer concentration with a low lipid concentration, the particle size significantly reduced, and vice versa. When the polymer Content was 1:2 with amount of lipid and the surfactant concentration was high, the reduction in particle size was more pronounced [50].

#### Zeta potential of lipid polymer hybrid nanoparticles

**Fig. 4:**
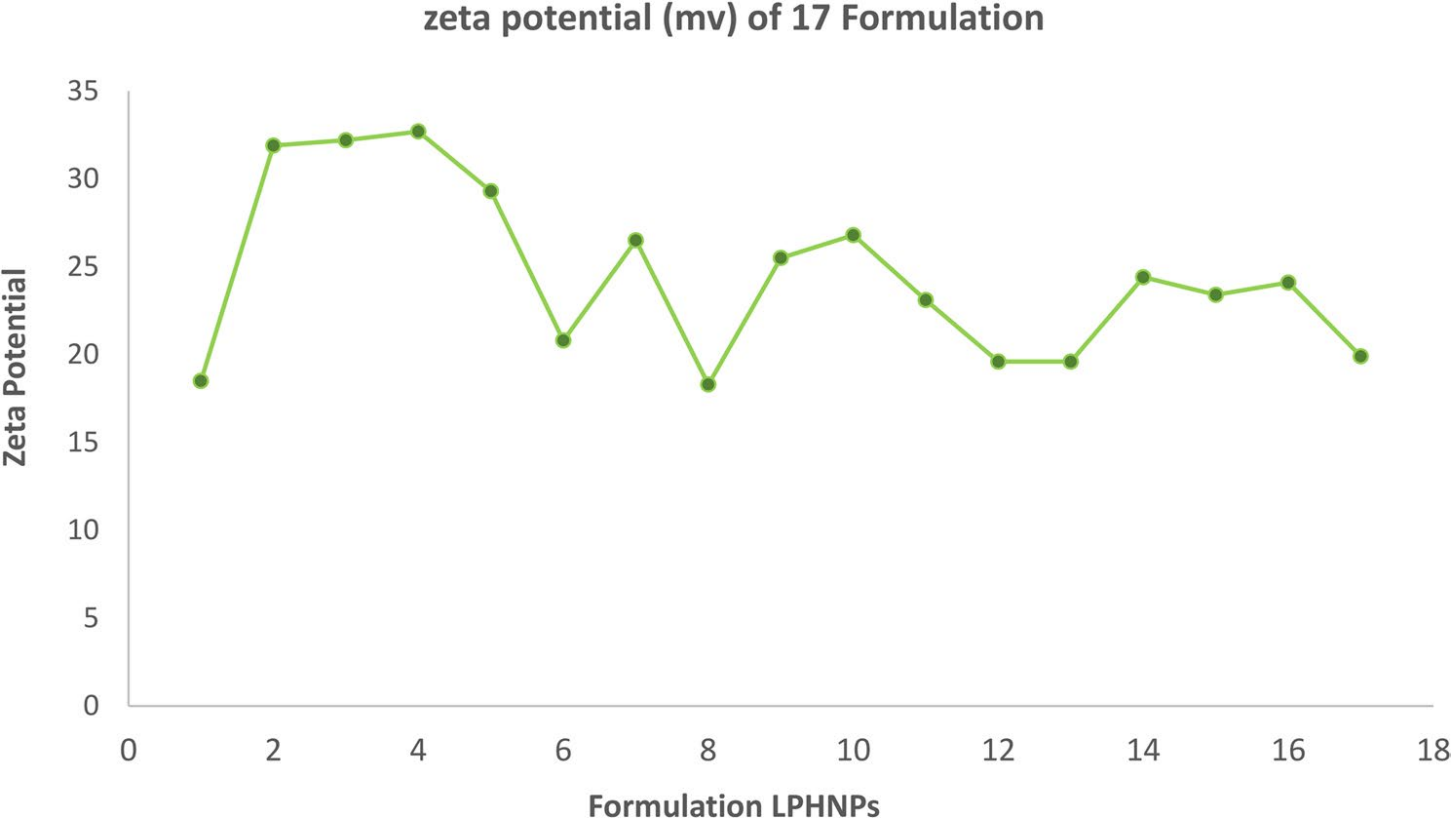
Variation in Zeta potential of 17 Nanoformulation

The zeta potential is the electrical charge at the hydrodynamic shear plane. The magnitude of the zeta potential indicates the electro-kinetic stability of colloidal dispersion and the intensity of the repulsive forces between the particles in the formulation. NPs are considered stable in general if their zeta potential is at least ± 30 mV. The negative zeta-potentials of all the formulations in our study ranged from − 18.5 to −32.7 mV, which is below (in certain formulations) the threshold range for pure electrostatic stabilization. The variation of zeta potential of different formulation is given in Fig. 4. However, surfactant and hydrophilic PEG impose electrostatic repulsion through steric stabilization on the surface of lipid polymer hybrid nanoparticles. This result was consistent with other work.

The ANOVA result indicated that the interaction impact of AB, BC, and CD, as well as surfactant concentration (C), were important determinants of this parameter. The zeta-potential of the particles was mostly influenced by the surfactant concentration. Consequently, increasing the surfactant concentration from 1 to 3.6% resulted in a considerable rise in the absolute zeta potential value. This could be because, as compared to bigger NPs, the smaller NPs exhibit a greater surface charge density [49]. In addition, the presence of surfactant contributes to surface charge, which enhances colloidal stability through electrostatic or steric repulsion, thereby improving the zeta potential values. Surfactant (*p*-value < 0.0001), polymer (*p*-value 0.03/0, lipid (*p*-value < 0.0001), stirring time (*p*-value < 0.0001).

#### (a) graphical analysis

For any formulation, the zeta potential is significantly impacted by four factors. It was depicted from data that was analyzed by design expert software. Normal plots of response surface model (RSM) showed that different concentration of polymer, lipid and surfactant affect zeta potential. The presence of surfactant contributes to surface charge, which enhances colloidal stability through electrostatic or steric repulsion, thereby improving the zeta potential values. Differences in zeta potential is clearly visible from graphical analysis. It can be seen with in Fig. 4. Zeta potential of all formulation is determined by central composite design (CCD). Differences in zeta potential of all formulation showed that different concentration of polymer, lipid and surfactant had big impact over it. Zeta potential was varied from − 18.3 to −32.7. Four independent components’ significant levels were determined using a central composite design. Multiple regression analysis and the model’s coefficient were used to assess the relevance of the four elements’ levels [50].

Four independent variables showed significant linear, interaction, and quadratic effects (*p* < 0.05). Particle size independent variables exhibited an excellent regression coefficient (R2) and were quadratic (0.996). Design Expert software version.13, which is provided below, was used to produce the final particle size prediction equation;

$$\begin{aligned}\bf\text{Y}1=&\bf-19.70-0.0173\text{A}-2.25\text{B}\\&\bf-2.08\text{C}-2.06\text{D}-1.34\text{AB}\\&\bf+0.1125\text{AC}+0.0340\text{AD}\\&\bf-0.5375\text{BC}-0.0405\text{BD}\\&\bf-0.0380\text{CD}+0.4458\text{A}^2\\&\bf-3.13\text{B}^2-3.34\text{C}^2-3.42\text{D}^2\end{aligned}$$

**Fig. 5:**
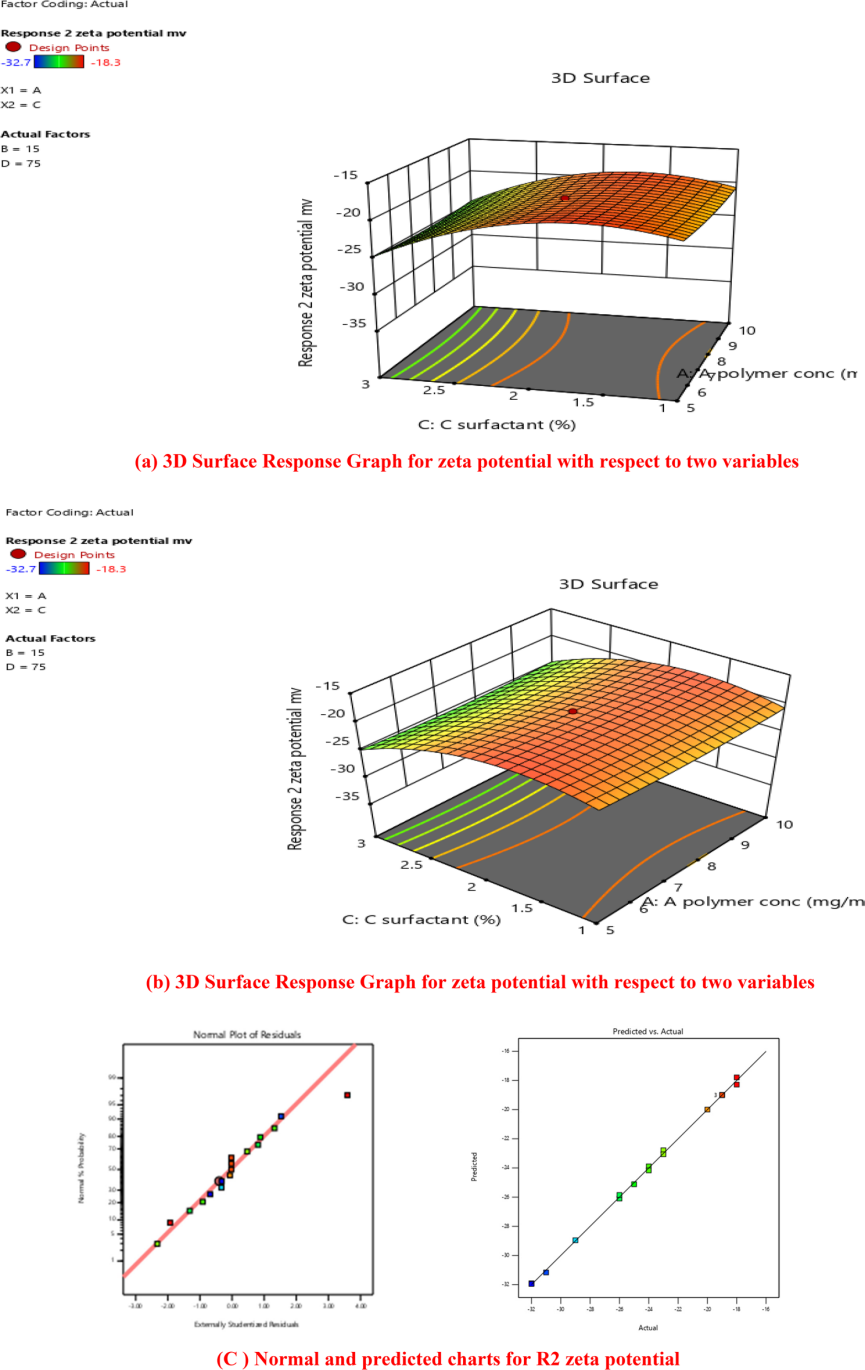
(**a**) 3D Surface Response Graph for zeta potential with respect to two variables. (**b**) 3D Surface Response Graph for zeta potential with respect to two variables. (**c**) Normal and predicted charts for R2 zeta potential

Three-dimensional response surface contour plots (surface response graphs) were used to depict the optimum level impacts of four independent factors on zeta potential. Four independent factors were shown to have a considerable impact on particle size [49]. It may be found in Fig. 5 (a) and (b).

For certain amounts of each element, predictions regarding the reaction may be made using the equation expressed in terms of coded factors. By default, the components’ high and low values are denoted by the numbers + 1 and − 1, respectively. By comparing the factor coefficients, the coded equation may be used to determine the relative importance of each component.

The relation between Factor A (polymer) and Factor C (surfactant) was given in 3D response surface plot. The plot clearly showed that, while operating at low polymer, there was a substantial variation in the zeta potential when going from low polymer (5 mg/ml) to high polymer (10 mg/ml) [49]. When operating at a high polymer concentration with a low surfactant percentage, the zeta potential significantly changed, and vice versa. When the polymer content along with surfactant concentration were changed, zeta potential was reported either high or low. Also, the lipid concentration and stirring time showed a visible effect on the zeta potential. For optimum zeta potential an optimum ratio of all variables was suggested by central composite design [50].

#### Regression coeffiicent an analysis of variance (ANOVA) for four responses

Regression Coefficient and ANOVA analysis was conducted based on the selected effects that were labeled in 3D and normal plots. The results of the ANOVA showed that the curvature was not significant. In the proposed model, quadratic model, had F-value and was highly significant for four responses and a P-value was less than 0.0001. The Lack of Fit F-value near to 1 showed that the model was not curvature-free. The descriptive statistics validate the model’s dependability. A high R-Squared value (R^2^ 0.8848) suggested that the appropriate equation adequately described the results of factorial experiment [49]. All findings from Analysis of Variance (ANOVA) and Regression Coefficient are given in Table 5.

The fact that there were sufficient level of freedom to adequately estimated the data allowed the Adjusted R-squared value (0.999) to be quite similar to the R-squared value. There is a fair amount of agreement between the adjusted R-squared and the predicted R-squared (0.990) of all responses. The experiment was done with a high degree of precision and dependability, as evidenced by the regression coefficient of variation [50].

#### Encapsulation efficiency of drug loaded lipid polymer hybrid nanoparticles

The % encapsulation efficiency results showed that the concentration of factor C (surfactant) significantly affects the nanoparticles’ encapsulation efficiency, which is supported by the low P-value of < 0.0012.

The surfactant and the drug’s physicochemical properties are the main factors that influencing the amount of drug entrapped in the nanocarrier. Table 4 showed that all formulations had good EEs, ranging from 49.9 to 83.3%, indicating that the high amount of drug was entrapped in the lipid polymer hybrid nanoparticles (LPHNPs).This result may be the result of drug lipophilic nature and low solubility in the external aqueous phase [50].

DL values% for prepared NPs range from 5.5 to 15.7% and were only influenced by the amount of polymer (A) used in each formulation. The DOE result showed that when the lipid content rose, the number of excipients increased as well, resulting in a fall in the DL%. An interacting impact of CD (surfactant concentration and stirring duration) was shown to be the most important element in the EE. The ANOVA result in Table 5.5 further demonstrated the noteworthy interaction between BC and AC on the EE of lipid polymer hybrid nanoparticles loaded with crizotinib [49].

#### (a) graphical analysis

**Fig. 6:**
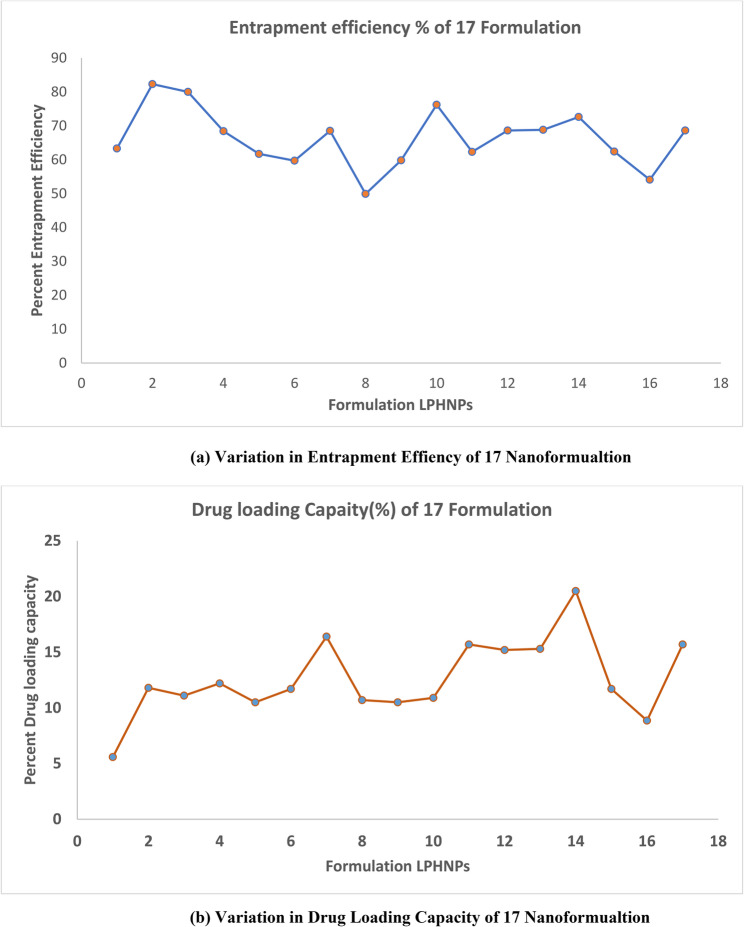
(**a**) Variation in Entrapment Effiency of 17 Nanoformualtion. **b** Variation in Drug Loading Capacity of 17 Nanoformualtion.

Among four variables the surfactant concentration has significant effect on encapsulation efficiency of all formulations. It was determined from data that was analyzed by design expert software. 3D plots of response surface model (RSM) showed that different concentration of surfactant, and polymer affect entrapment efficiency. Polymer concentration demonstrated a positive correlation with particle size. A higher amount of polymer increases the viscosity of the organic phase, thereby restricting efficient droplet breakdown and leading to the formation of larger particles. At the same time, a higher polymer content favors drug entrapment efficiency due to the greater availability of matrix material for drug accommodation. However, excessive polymer may lead to irregular morphology and less uniform distribution. Thus, polymer concentration exerted both linear and quadratic effects on nanoparticle size and encapsulation efficiency. Differences in entrapment efficiency is clearly visible from normal plot as shown in Fig. 6 (a).

Every formulation’s entrapment efficiency is based on central composite design (CCD). Variations in the entrapment efficiency across all formulations demonstrated that surfactant, lipid, and polymer concentrations had a significant influence on it. The range of entrapment efficiency was 49.9 to 83.3.

Using a central composite design, the significant values of four independent components were determined. The significance of the four component levels was evaluated using multiple regression analysis and the model’s coefficient [49]. Surfactant (*p*-value < 0.0001), polymer (*p*-value < 0.0001), lipid (*p*-value < 0.0001) indicates significance effects on responses.

The four independent variables’ linear, interaction, and quadratic impacts were shown to be significantly different (*p* < 0.05). Entrapment efficiency, independent variables had an excellent regression coefficient R^2^ (0.998), being quadratic. The design expert software version.13 was used to produce the final prediction equation for entrapment efficiency;

$$\begin{aligned}\bf\text{Y}1=&\bf68.64-0.2658\text{A}+0.1485\text{B}\\&\bf+0.1596\text{C}+0.0059\text{D}\\&\bf+0.03637\text{AB}-0.0265\text{AC}\\&\bf+0.0012\text{AD}+0.0221\text{BC}\\&\bf+0.0432\text{BD}-0.0214\text{CD}\\&\bf-0.2749\text{A}^2+0.1919\text{B}^2\\&\bf-0.1011\text{C}^2-0.0032\text{D}^2\end{aligned}$$

**Fig. 7:**
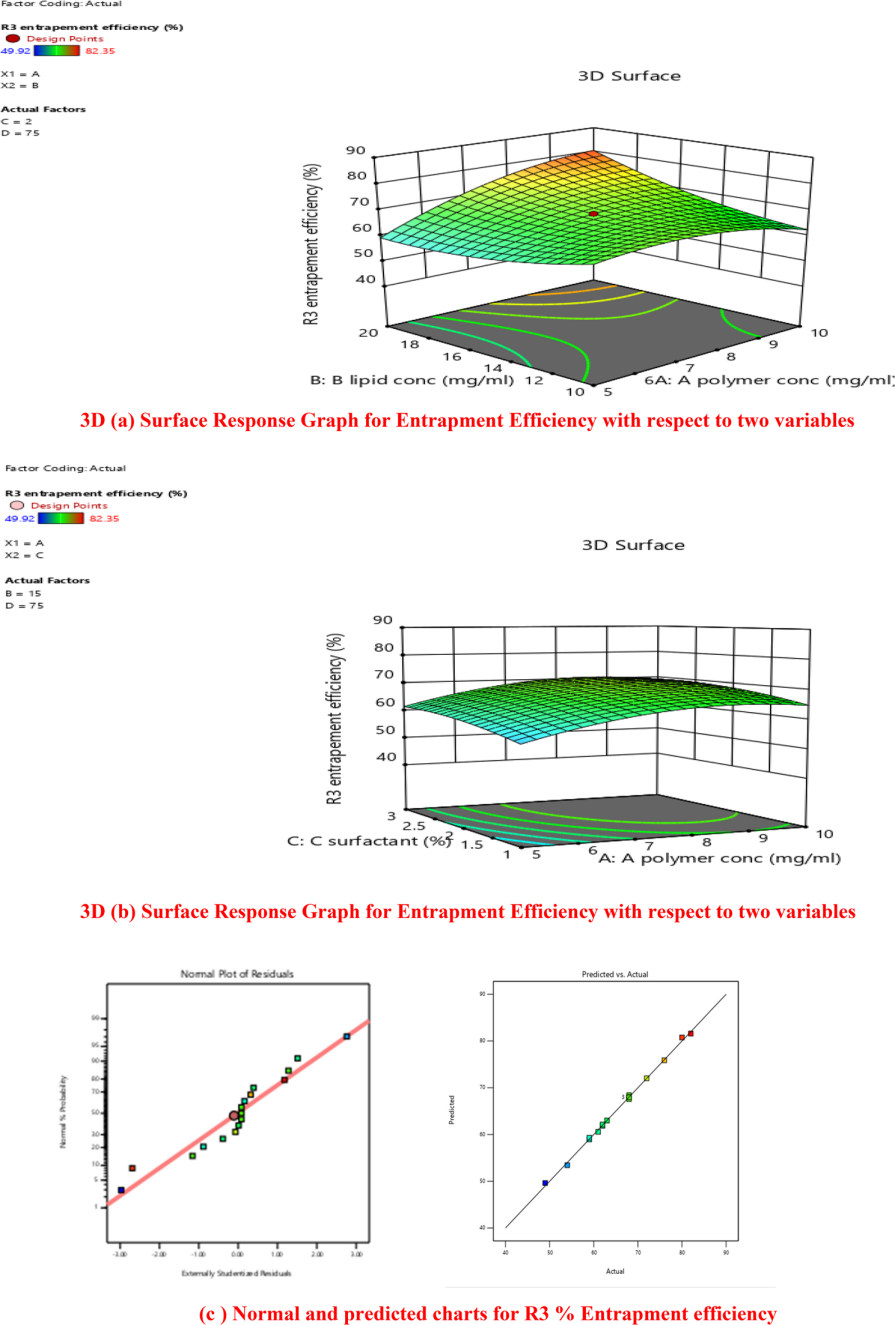
3D(**a**) Surface Response Graph for Entrapment Efficiency with respect to two variables. 3D (**b**) Surface Response Graph for Entrapment Efficiency with respect to two variables. (**c**) Normal and predicted charts for R3% Entrapment efficiency

Three-dimensional response surface contour plots, also known as surface response graphs, were used to explain the optimum level impacts of four independent factors on entrapment efficiency. Four independent factors were shown to have a substantial impact on entrapment efficiency. As seen in Fig. 7 (a) and (b).

The equation in terms of coded factors can be used to predict the reaction for certain concentrations of each ingredient. The high and low values of the components are automatically represented by + 1 and − 1, respectively. The relative relevance of each component may be ascertained by utilizing the coded equation to compare the factor coefficients [49].

A three-dimensional response surface plot was used to show the interaction between factor A (polymer) and Factor C (surfactant). The figure made it abundantly evident that there was a significant change in the entrapment efficiency of formulation while operating at low polymer (5 mg/ml) compared to high polymer (15 mg/ml). The encapsulation efficiency altered dramatically while operating at a low surfactant % with a high polymer concentration, and vice versa. It was reported that formulation with high concentration of surfactant with high amount of polymer gave maximum entrapment efficiency. There was also a discernible impact of the stirring duration and lipid content on the entrapment efficiency. The central composite design proposed an ideal ratio of all factors for getting an optimal level of entrapment efficiency [49].

#### Effect of factors on drug release from drug loaded lipid polymer hybrid nanoparticles

To provide a sink environment, ex vivo release experiments were carried out in PBS with 2% Tween80. Every profile had a burst release in the early incubation hours, which is connected to the drug on outside of the LPHNPs. Subsequently, a sustained discharge of drug occurred as a result of the drug’s diffusion within the NPs’ core. It was observed that biphasic release patterns had present [49]. To correlate the various profiles of drug release for produced nano-formulations, central composite design was used. Table 4 illustrated drug release from different formulation was fluctuated between 42.7% and 60.6% in 12 h.

The amount of polymer (A), lipid concentration (B), surfactant concentration (C), stirring duration (D), and the total impact of AB, AC and CD were the key parameters that substantially affected the in vitro drug release. The lipid Content also significantly affected the nanoparticle properties. Lipid constitutes the outer shell of the hybrid system, and an insufficient amount may result in incomplete surface coverage of the polymeric cores, causing particle aggregation, increased PDI, and reduced stability. In contrast, an adequate lipid-to-polymer ratio provides a uniform coating that enhances colloidal stability and minimizes drug leakage. However, a very high lipid concentration may lead to particle aggregation or modification of drug release behavior. The release data analysis indicated that the AC interaction had the greatest impact on the drug release. The outcome shown that increasing the stirring period from 30min to 120 min and surfactant concentration led to a higher drug release percentage, as shown by the Table 4.

It is also demonstrated that particle size had decreased as a result of both of them. The size of particles has a important effect on the release rate of nanoparticles. The release rate of nanoparticles was shown to be inversely correlated with their particle size. When the particle size was decreased the release rate of the drug was increased. It is also supported by the fact that smaller particles have shorter diffusion paths and a greater surface area/volume ratio. So, greater drug fraction exposure to the release media resulted from this. On the other hand, larger particles and a slower rate of drug release were produced by increasing the lipid concentration and minimum polymer and surfactant concentration. Additionally, increases in surfactant concentration would had improved crizotinib solubility in the aqueous phase and accelerate medication diffusion there [49].

#### (a) graphical analysis

The amount of polymer and surfactant content are two of four factors that significantly affects the drug release. It was ascertained by the use of design expert software to examine data. Response surface model (RSM) normal plots demonstrated how in vitro drug release is influenced by surfactant and polymer concentrations. Variations in drug release from different formulation was easily discernible from 3D plot.

Central composite design is the foundation for each formulation’s entrapment efficiency (CCD). Surfactant, lipid, and polymer concentrations were shown to have a substantial effect on the in vitro drug release, as seen by variations in this parameter across all formulations. The in vitro drug release had ranged from 42.7 to 60.6%.

The significant values of four independent components were ascertained by use of a central composite design. The significant levels for the four components was evaluated using multiple regression analysis and the coefficient of model.

The four independent factors’ linear, interaction, and quadratic effects varied significantly (*p* < 0.05). The independent factors pertaining to entrapment efficiency exhibited a quadratic regression coefficient of good R2 (0.999). The p-value for different variables polymer (*p*-value 0.0028, lipid (*p*-value 0.013) surfactant (*p*-value 0.017) show significance effect on in vitro drug release.

The final in vitro drug release prediction equation was created using the Design Expert software version 13.

$$\begin{aligned}\bf\text{Y}1=&\bf 56.23+0.1356\text{A}+0.1357\text{B}\\&\bf+0.0070\text{C}+0.0386\text{D}+0.0485\text{AB}\\&\bf+0.2318\text{AC}+0.2154\text{AD}-0.0364\text{BC}\\&\bf+0.1926\text{BD}-0.2911\text{CD}-0.1888\text{A}^2\\&\bf+0.0557\text{B}^2-0.2133\text{C}^2-0.0293\text{D}^2\end{aligned}$$

**Fig. 8:**
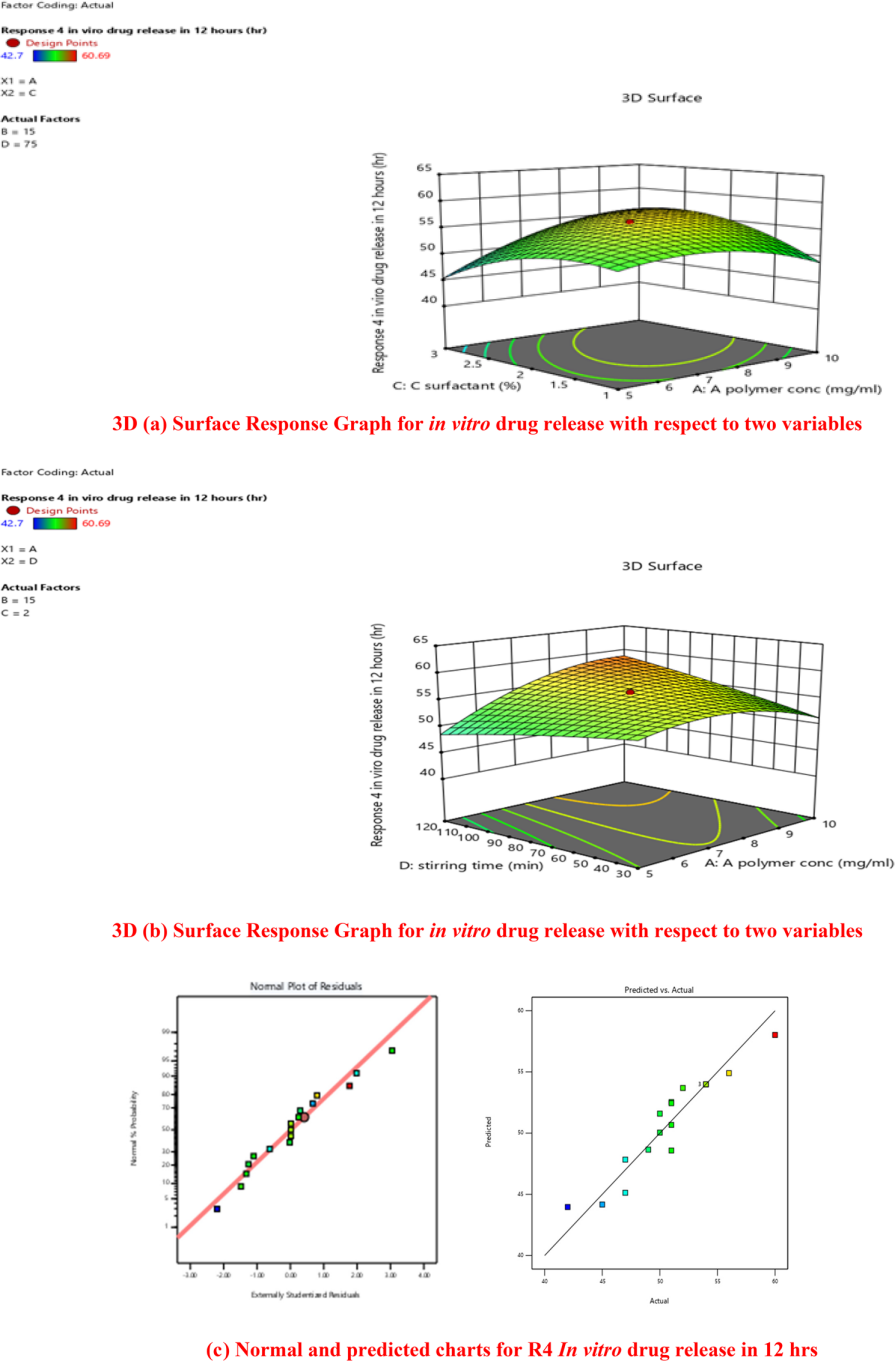
3D (**a**) Surface Response Graph for in vitro drug release with respect to two variables. 3D (**b**) Surface Response Graph for in vitro drug release with respect to two variables. (**c**) Normal and predicted charts for R4 In vitro drug release in 12 h

3D Surface response graphs, or three-dimensional response surface contour plots, were employed to elucidate the optimal level effects of four separate parameters on ex vivo release of drug. The in vitro release of drug was shown to be significantly influenced by four independent parameters. As seen in Fig. 8 (a) and (b).

For certain concentrations of each component, the reaction may be predicted using the equation expressed in terms of coded factors. The components’ high and low values are automatically rendered as + 1 and − 1, respectively. By using the coded equation and comparing the factor coefficient, one may determine the relative importance of each component [49].

**Fig. 9:**
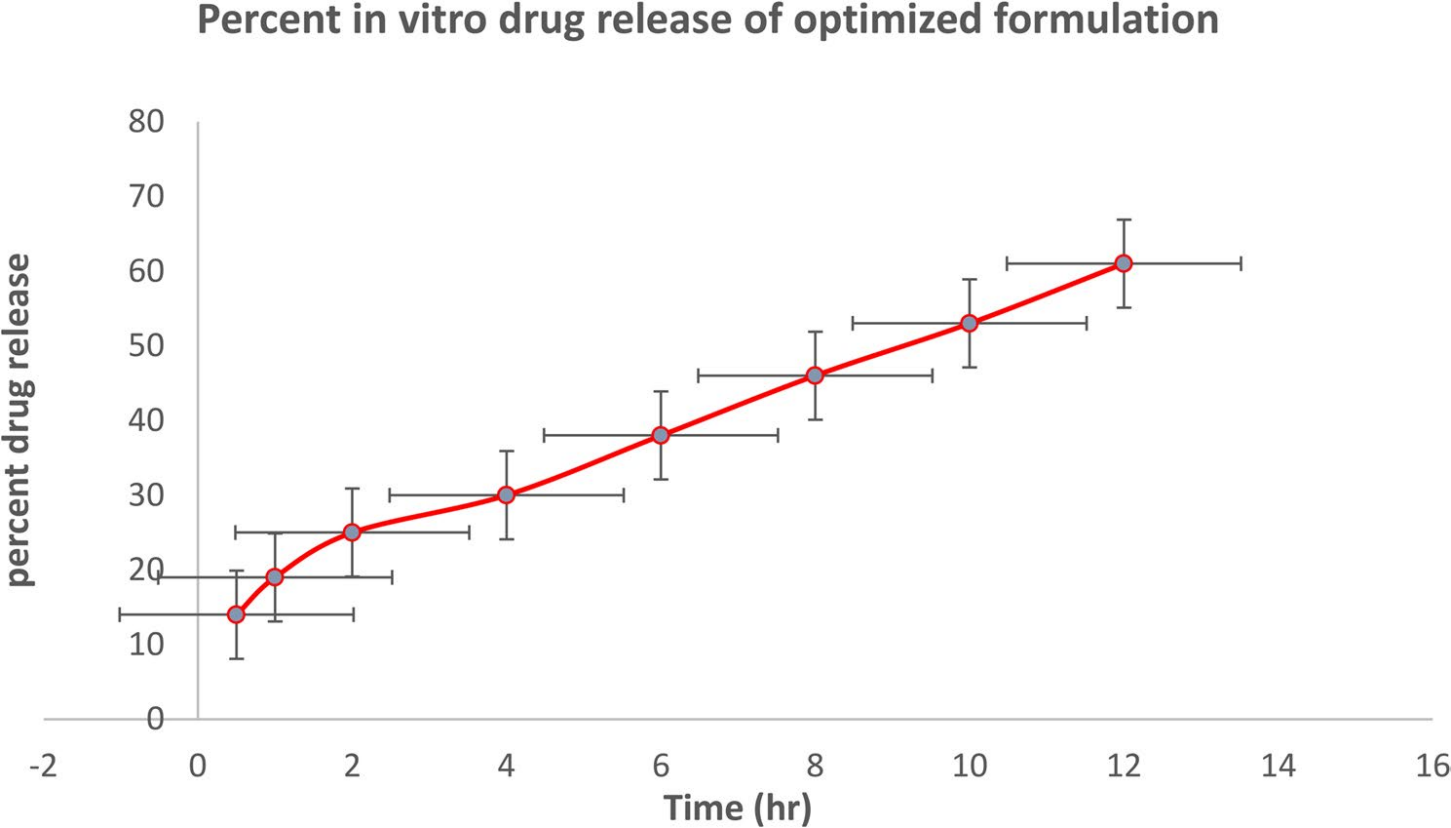
Study of Optimized Formulation for in vitro Drug Release

Factor A (polymer) and Factor C (surfactant) interacted, as seen by a Response surface plot in three dimensions in Figure.8 (a). The figure clearly showed that the formulation’s in vitro drug release had changed significantly while it was running at a low polymer (5 mg/ml) as opposed to a high polymer (15 mg/ml). When operating at a low surfactant percentage with a high polymer concentration, the in vitro drug release changed significantly, and vice versa. Maximum in vitro drug release was recorded when a formulation with high amount of surfactant and a high quantity of polymer was used. It was also shown in 3D surface response plot in Fig. 8 (b) that interaction of factor A and factor D had impact on in vitro drug release [49]. The lipid content with stirring time had a noticeable effect on in vitro drug release as well. To achieve the best possible degree of ex vivo drug release, the central composite design suggested the appropriate ratio of all the elements. Drug release for optimized formulation F2 that has shown maximum release with respect to time is given in Fig. 9.

#### In-Vitro release kinetics

Several pharmacokinetic models, such as zero order, first order, Higuchi, Hixson and Crowell, and Korsemeyer models, were fitted to the data in order to assess the kinetics and predict the probable drug release mechanism from the drug loaded lipid polymer hybrid nanoparticles. The results are shown in Table 6.

**Table 6:** Percent accumulative release of drug loaded lipid polymer hybrid nanoparticles of optimized formulation

S. No	Time (Hr)	% Cumulative release	Kinetic Models and *R*^2^ value
1	0.5	14 *±* 0.816	Zero order R^2^
2	1	19 *±* 1.24	0.889
3	2	25 *±* 1.30	First order R^2^
4	4	30 *±* 1.27	0.9915
5	6	38 *±* 1.23	Higuchi model R^2^
6	8	46 *±* 0.24	0.983
7	10	53 *±* 0.812	Korsmeyar-peppas model R^2^
8	12	60.6 *±* 0.6	0.893

**Fig. 10:**
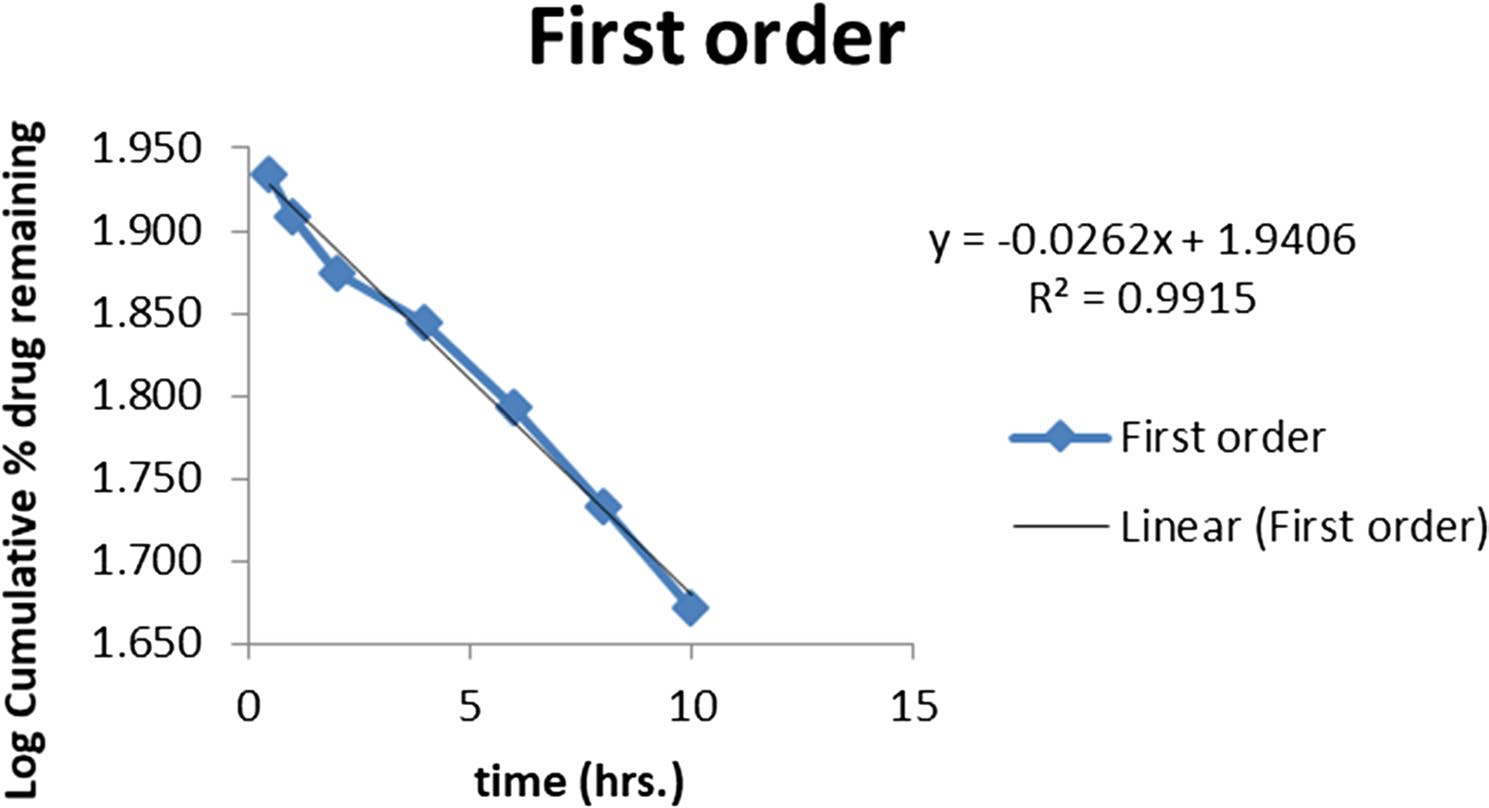
Percent Cumulative Drug release of Optimized Formulation

The zero-order kinetics’ R2 value of 0.889 demonstrated that the drug is released gradually, the dissolution from lipid polymer hybrid nanoparticles is concentration-independent, and they do not disintegrate. R2 value of 0.9915 demonstrated first order kinetics that means drug release is dependent of concentration available. It is shown in Fig. 10. In order to forecast a biphasic release pattern, the data of drug-loaded polymeric nanoparticles was evaluated using a single-phase method to drug release. The high R^2^ value of 0.983 from the single-phase method study showed that the Higuchi model best fits the data. The Higuchi model verifies that the drug release occurs via a diffusion mechanism that is square root time dependent and based on Fick’s law.

This in vitro release study also demonstrated distinct differences between crizotinib-loaded nanoparticles and the free drug solution. While the free drug exhibited an initial burst release, with more than 80% released within the first 2 h, the nanoparticle formulation showed a controlled and sustained release profile, with approximately 80% released over 24 h. This sustained release behavior can be attributed to the encapsulation of crizotinib within the polymer–lipid hybrid matrix, which provides diffusional resistance and controlled drug diffusion. Such a release pattern underscores the potential of the formulation to achieve prolonged therapeutic concentrations in the lungs, thereby reducing dosing frequency and improving patient compliance.

#### Verification of predicted model

Different experiments verified the predicted model to be designed. The suggested ratio of four variables (polymer conc. lipid conc. %Surfactant and stirring time) were applied for preparation of lipid polymer hybrid nanoparticles. The outcomes were identical to what the expected model indicated.

Table 7 had shown the predicted levels of factors that the central composite design proposed to get the best four responses for optimized lipid polymer hybrid nanoparticles.

**Table 7:** Estimated and tested values for four answers

Responses	Appropriate level of polymer (mg/ml)	Desirable value of lipid (mg/ml)	Maximum ratio of surfactant (%)	Optimum ratio of stirring time (min)	Predicted value	Experimental value
Particle size	10 mg/ml	20 mg/ml	3%	116.97	161.87 *±* 0.37	167.2 ± 17.7
Zeta potential	10 mg/ml	20 mg/ml	3%	116.97	−31.8 *±* 0.1	−32.3 ± 0.7
Entrapment efficiency	10 mg/ml	20 mg/ml	3%	116.97	81.6 *±* 0.33	82.3 ± 0.05
In-*vitro* drug release in 12 h	10 mg/ml	20 mg/ml	3%	116.97	60.69 *±* 0.28	60.6 ± 0.6

The design expert software also recommended an anticipated ratio of all excipients concentration in nano-formulation in order to obtain a nano-formulation that can provide positive outcomes.

**Fig. 11:**
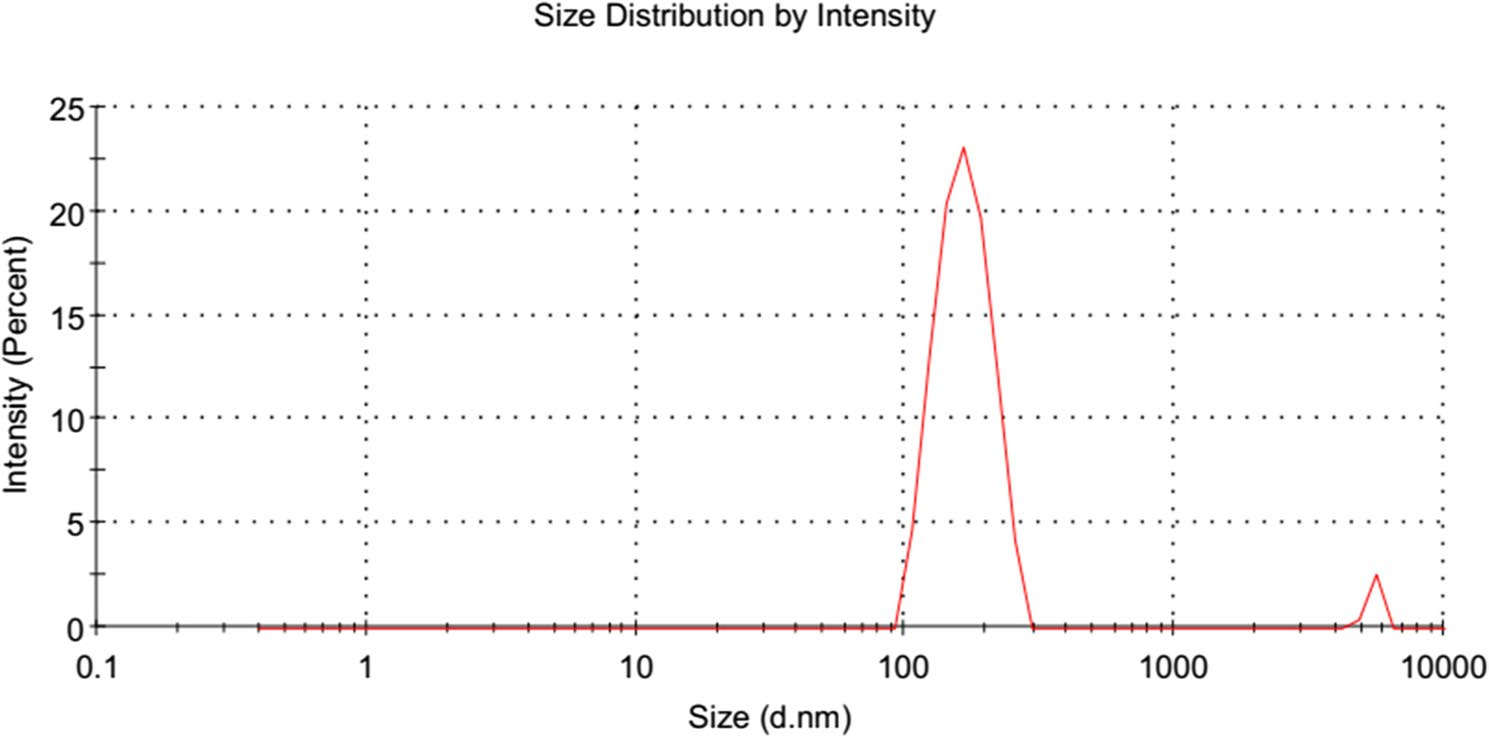
The optimized drug-loaded LPHNPs’ size and PDI graph

**Fig. 12:**
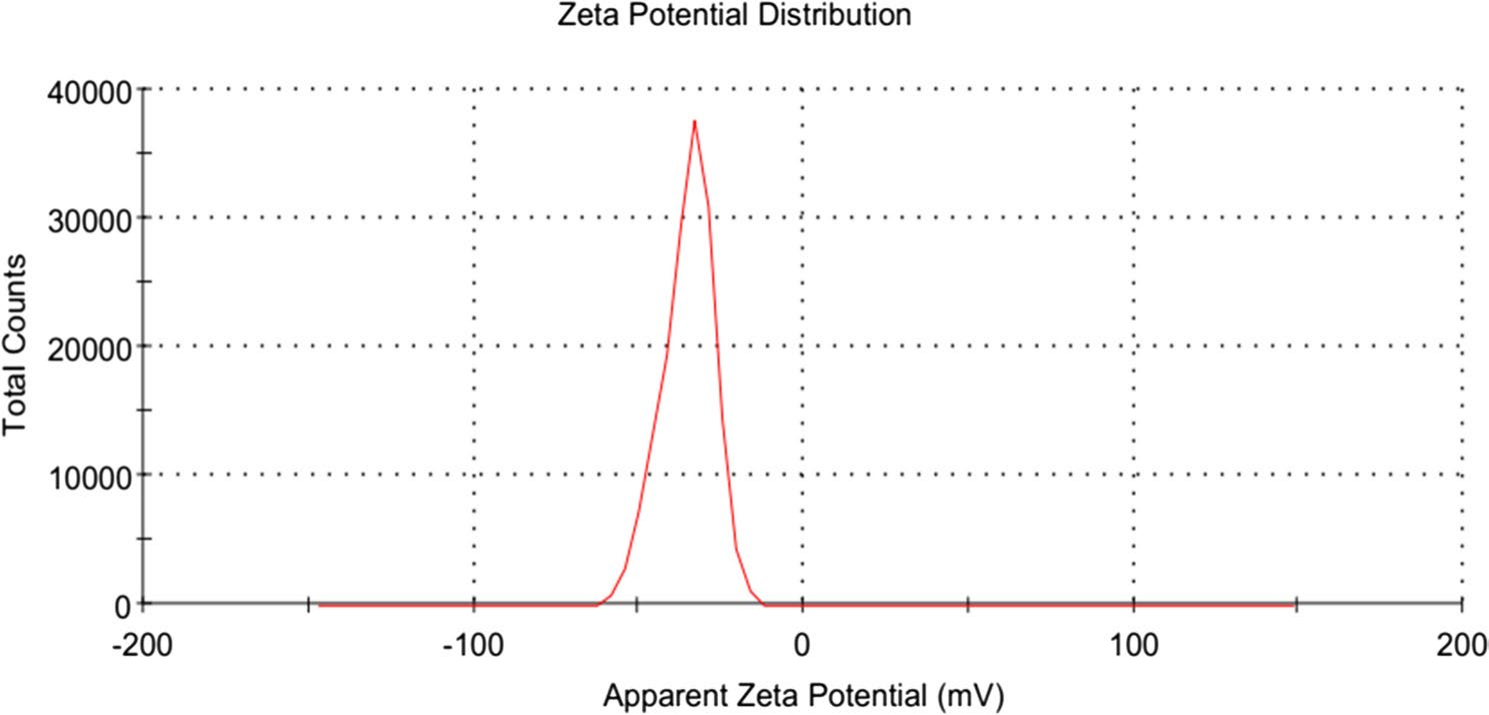
Optimized Zeta Potential Graph of Drug-Loaded LPHNPs

Because of the projected model’s minimum particle size, targeted zeta potential, high encapsulation efficiency, and maximum in vitro drug release in 12 h an optimized formulation was made in accordance with it [50]. Minimum particle size and optimum zeta potential for an optimized formulation is shown in Figs. 11 and 12.

The experiment was carried out using the anticipated model in accordance with the software’s recommended ratio. Test results confirmed the model’s prediction. Lipid polymer hybrid nanoparticles were prepared using the indicated concentration of all variables. The same outcomes were attained that confirmed the correct use of the model.

The experiment’s identical outcomes validated RSM i.e. central composite design for lipid polymer hybrid nanoparticles preparation. The experiment’s results verified that there was no discernible difference between the expected and actual outcomes. To put it briefly, this model may be utilized to optimize the amount of polymer, lipid and surfactant, while making lipid polymer hybrid nanoparticles of crizotinib [50–55].

### Characterization results of lipid polymer hybrid nanoparticles

#### Scanning electron microscopy (SEM)

**Fig. 13:**
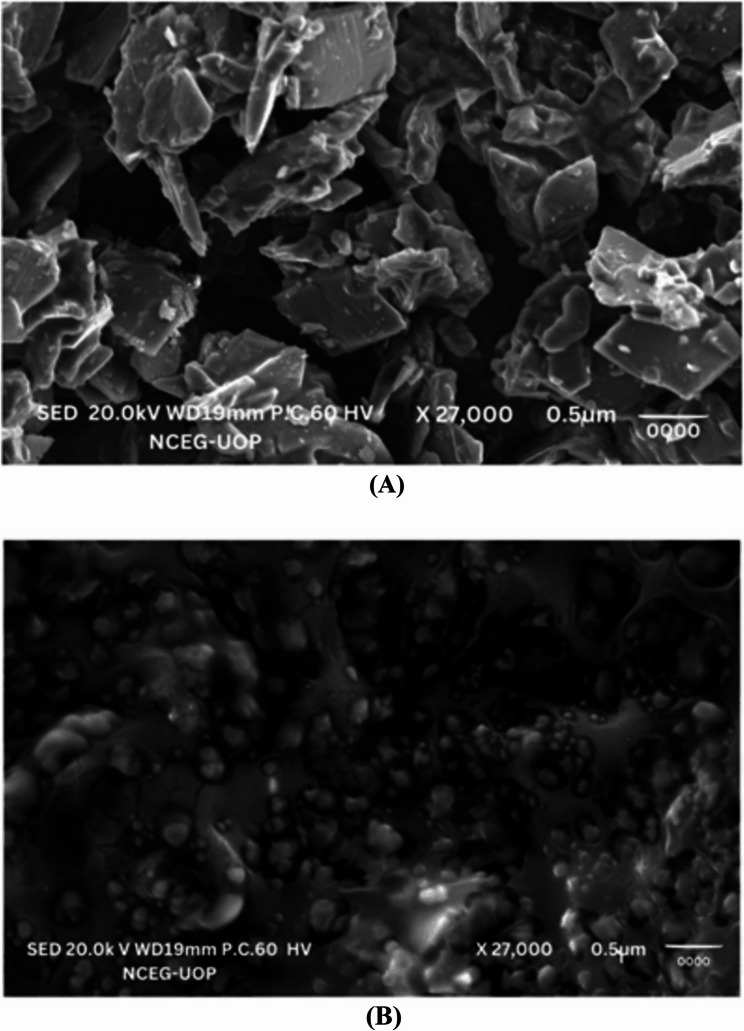
(**a**) SEM image of pure drug. (**b**) SEM image of LPHNPs.

The SEM image of the pure drug was examined for the purpose of visual evaluation of the crizotinib. The crystalline, cylindrical structure of the pure drug nanoparticles with smooth surfaces is seen in the SEM picture in Fig. 13(A).

The optimized nano- formulation’s SEM picture amply demonstrated that the spherical-shaped LPHNPs had smooth surfaces and were less than 200 nm. Although there were occasional aggregations, LPHNPs were primarily distributed. The SEM image of LPHNPs can be seen in Fig. 13(B).

#### Powder X-ray diffraction

**Fig. 14:**
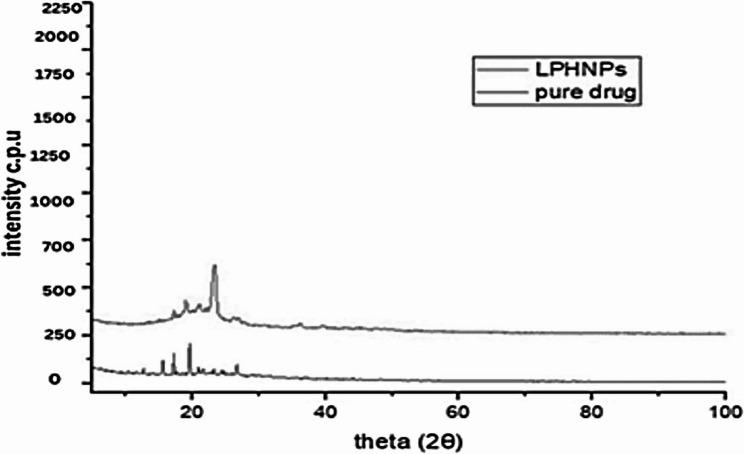
XRD Spectra for Pure Drug (-) and LPHNPs (-)

When comparing the adjusted optimized nano formulation to crizotinib (pure drug), the powder X-ray diffraction pattern revealed strong peaks with increased peak counts at the top that can be seen in Fig. 14 with black demarcated line. The improved optimized nano formulation’s diffused peaks in the P-XRD pattern in Fig. 14 with pink demarcated line showed that it was amorphous. For the drug (crizotinib) to transition to an amorphous state, reduced mean particle size, greater surface area, and robust Tween-80(surfactant) -drug interaction might be taken into consideration.

#### Differential scanning calorimetry

A DSC investigation was carried out to find out the melting points of the components that went into making the LPHNs. DSC analyses were carried out on raw/pure Crizotinib, and processed Crizotinib treated with polymer and lipid.

**Fig. 15:**
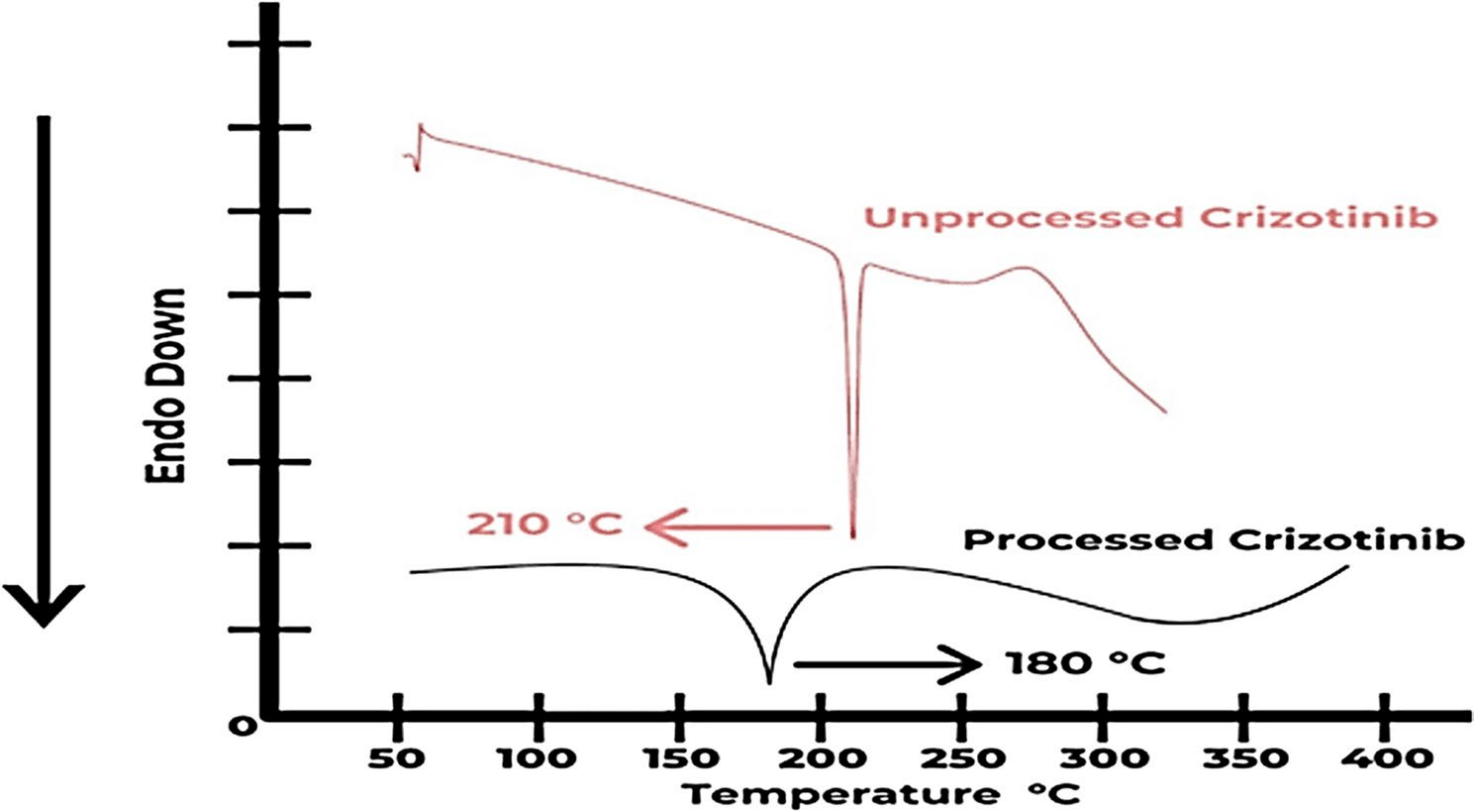
DSC Spectra for Unprocessed Drug and Processed Drug

The processed Crizotinib-loaded LPHNPs’ DSC thermos-gram in Figure. 15 clearly displayed melting point peaks for the supporting lipids and polymers. For processed Crizotinib LPHNPs, a very tiny and wider endothermic peak had emerged, indicating a significant miscibility of the drug with excipients and development towards an amorphous state.

At 210 °C, the melting point maxima for unprocessed Crizotinib was observed. On the other hand, the melting point peaks was somewhat lowered at 180 °C, for the generated LPHNs of Crizotinib. The generated LPHNs’ wider peaks and little drop in the melting point peaks amply illustrated the proper miscibility of drug with excipients.

#### Fourier Transform Infra-Red spectroscopy (ftir)

**Fig. 16:**
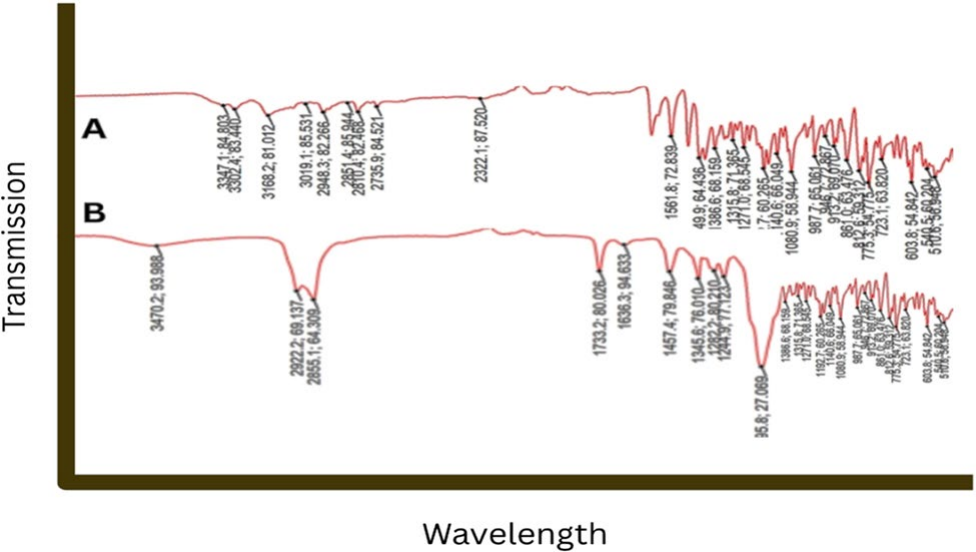
FTIR Spectra for Pure Drug (**A**) and LPHNPs (**B**)

Using the KBr disc technique (PerkinElmer, Waltham, MA, USA), FTIR spectroscopy was used to investigate any interactions that could exist between the Crizotinib, polymer, and other excipients in the optimized LPHNPs formulation. A spectral range of 4000–500 cm was used to record the transmittance (%T). All of the main peaks are visible in the FTIR spectrum Figure. 16 (A) which verified that the Crizotinib had maintained its structure. The aromatic compound at 840 cm-1, the C-N stretch at 1242 cm-1, the ketone group at 1762 cm-1, the C = C stretching at 1604 cm-1, and the OH group at 4004 cm-1 were the main peaks that were seen. There were slight peak shifts and broadenings that might have been caused by a physical interaction between the medication and the excipients in LPHNPs. It is shown in Fig. 16 (B) spectra.

### Stability studies

Stability tests were carried out on the optimized lipid polymer hybrid nanoparticles (LPHNPs) nano-formulations at two different temperatures, i.e., at room temperature (25 °C) and refrigerated temperature (4 °C), in order to evaluate the impact of storage on the PS, PDI, and EE of the nanoparticles. To test the stability, of nano-formulation, when compared to room temperature, where the changes were considerable, the noticed modification in size of particles, PDI, and %EE at 4 °C were not statistically significant. The findings show that, in contrast to high temperatures, which encourage particle aggregation, low temperatures discourage particle aggregation because they have lower kinetic energy and hence inhibit particle collisions.

The findings indicated that the nano-formulations should be refrigerated at 4 °C for physico-chemical stability. Table 8 shows stability study for nanoformulation of crizotinib.

**Table 8:** Stability studies of optimized drug loaded LPHNPs stored at 4 °C and 25 °C

Time	NPs	Stored at 4 °C				Stored at 25 °C			
		PS	PDI	Zeta	%EE	PS	PDI	Zeta	%EE
Day 1	Drug loaded LPHNPs	167.2 ± 0.04	0.462 ± 0.04	−32.2 ± 0.09	82.3 ± 0.05	167.3 ± 0.11	0.462 ± 0.01	−32.2 ± 0.15	82.3 ± 0.05
Week 1		167.4 ± 0.16	0.461 ± 0.08	−32.0 ± 0.016	82.2 ± 0.07	183.3 ± 0.2	0.450 ± 0.02	−31.4 ± 0.20	81.6 ± 0.02
Week 2		167.2 ± 0.12	0.432 ± 0.03	−31.0 ± 0.20	81.6 ± 0.03	188.3 ± 0.58	0.462 ± 0.02	−29.0 ± 0.15	80.7 ± 0.23
Week 3		166.5 ± 0.12	0.455 ± 0.08	−29.8 ± 0.50	82.3 ± 0.01	192.5 ± 1.15	0.370 ± 0.01	25.7-±0.23	80.3 ± 0.33
Week 4		166.2 ± 0.65	0.449 ± 0.04	−29.8 ± 0.28	82.1 ± 0.02	207.1 ± 0.41	0.39 ± 0.01	−21.3 ± 0.25	79.4 ± 0.01

### Aerosolization and inhalation capabilities of Crizotinib nanoparticles

Eight crizotinib nanoparticles/Mannitol microparticles combinations by weight ratio of 1:1 were produced and passed to cascade impaction analysis (ACI). Two particle size levels were used to collect the respirable fractions and fine particles: less than 3 μm, and less than 5 μm. The first level represented that particles size is appropriated for deep lung accumulation. Particles with the optimal aerodynamic diameter for delivery to peripheral lungs were found in the second stage. Level three was the result of collection of those particles with a diameter of less than 0.5 μm, which are likely to be inhaled from the second level [35].

Optimized formulation of crizotinib nanoparticles with particle size 167 nm with physical admixture with mannitol showed higher DD, PD, PI, FPD, FPF and RF. That are mentioned in Table 9.

**Table 9:** Aerodynamic performance of Crizotinib loaded nanoparticles using ACI

Parameter	Crizotinib NPs
TD (mg)	14.432
ED (mg)	12.275 ± 1.145
ED%	>80
DD (mg)	11.449 ± 1.113
PD (%)	85.052 ± 2.002
PI (%)	79.331 ± 1.783
MMAD (µm)	1.515 ± 0.312
GSD	2.848 ± 0.126
FPF_≤3 μm_	56.218 ± 1.180
RF	60.274 ± 1.452
FPF_≤5 μm_	69.382 ± 1.706
RF	74.386 ± 1.958

The fact that the ED% was > 80% suggests that there was little medication loss in the capsule and device. The optimized nanoparticles with mannitol was an improved formulation had the lowest GSD and MMAD and the greatest FPF. One well-known factor affecting the location and mass of inhaled crizotinib deposited in the lung is the diameter of the particle which resides in aerodynamic range. Particles with diameter of between 1 and 5 μm have been demonstrated to reside deeply in the lung when used as therapeutic aerosols [35]. The mouth, nose, throat, and larynx have already accumulated the bigger particles. In contrast, the aerodynamic diameter of particulate matter less than 1 μm is mostly expelled, resulting in little particle deposition.

With mannitol, the MMAD of the spray-dried particles was 1.515 μm, suggesting that they were successfully deposited in the lungs. Through the DPI, the GSD shows the variation in particle dimensions. Broad size distribution, indicated by a lower GSD number, ensures consistent and repeatable therapy effects. Table 9 illustrated that the powder based on mannitol exhibited polydispersity in aerodynamic range, with a GSD of 2.48. Therefore, it was determined that the mannitol-containing formulation was the most suitable for MMAD, GSD, and FPF [35].

**Fig. 17:**
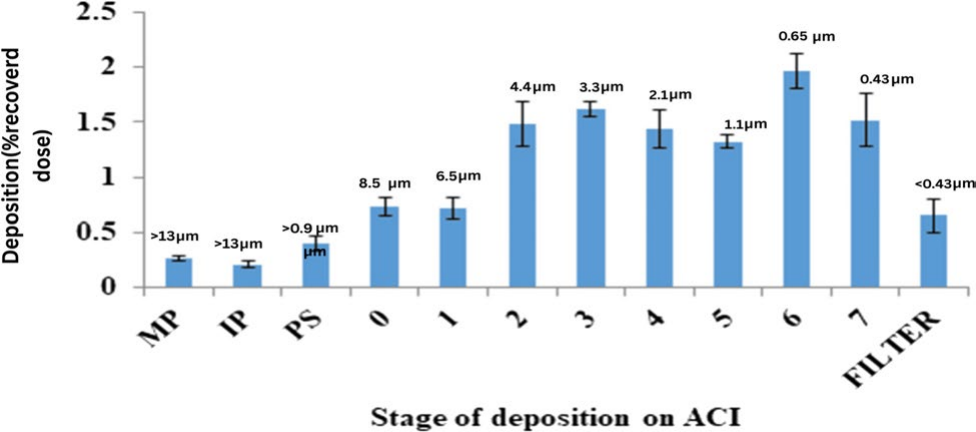
Deposition pattern of aerosol mass on different stages of ACI

Figure 17 also clearly showed that crizotinib nanoparticles are adsorbed effectively on Mannitol microparticles due to which their deposition in deep lungs and peripheral region was higher. From stage 0 to 7 the drug deposition in different parts of lungs was varied, that is shown in Fig. 17, but it was higher at stage 6 which showed that crizotinib nanoparticles were highly affected on deep lung non-small cell carcinoma. Crizotinib nanoparticles also perfectly hit peripheral and deep down areas of the lung.

## Conclusion

In this study, a mannitol-mixed dry powder inhalation (DPI) formulation containing Crizotinib lipid and polymer was successfully synthesized. The aerosol performance of the prepared dry powder formulation demonstrated promising characteristics, with a mass median aerodynamic diameter (MMAD) of 1.515 μm, an emitted dose (ED) of 12.275 mg, and a fine particle fraction (FPF) of 56.218%. Crizotinib-loaded nanoparticles were created using the nano-precipitation method. To optimize various formulation parameters including lipid ratio (mg/ml), polymer ratio (mg/ml), surfactant concentration (%), and stirring duration (min) a central composite design approach was employed. The characterization of the Crizotinib-loaded nanoparticles was performed using scanning electron microscopy (SEM), X-ray diffraction (XRD), and differential scanning calorimetry (DSC) to confirm the conversion to an amorphous phase. Key factors for the transition of Crizotinib to an amorphous state included a reduced average particle size, increased surface area, and strong interactions between the surfactant Tween-80 and the drug.

Fourier-transform infrared spectroscopy (FTIR) analysis was conducted to assess the relationship between Crizotinib and the excipients, indicating no significant interaction. In vitro studies provided strong evidence that the new Crizotinib formulation is effective for lung cancer treatment due to its favorable physicochemical characteristics and sustained release behavior, supporting their potential as an inhalable delivery system for lung cancer therapy. While the present study establishes proof-of-concept at the in vitro level, further in vivo investigations are required to evaluate pulmonary deposition, pharmacokinetics, antitumor efficacy, and long-term safety. These studies will be essential to confirm the therapeutic benefits observed in vitro and to optimize dosing regimens. Looking forward, the integration of in vivo data with translational research, including scalable manufacturing, regulatory considerations, and clinical validation, will be critical steps toward advancing this formulation from bench to bedside. The results from these experiments support the potential use of this formulation as a viable treatment option for lung cancer, paving the way for further clinical evaluation.

## Data Availability

Availability of data and materials: Yasar Shah.
